# Supplementary material for: Divergent molecular pathways govern temperature-dependent wheat stem rust resistance genes
Source: Nat Commun. 2025 May 28;16:4905. doi: 10.1038/s41467-025-60030-x (PMC12119863; doi:10.1038/s41467-025-60030-x)
Supplement: Supplementary file 1 — Supplementary Information [file 41467_2025_60030_MOESM1_ESM.pdf]

Divergent molecular pathways govern temperature-dependent wheat stem rust resistance genes

Hewitt, Sharma, and Zhang *et al.*

A)

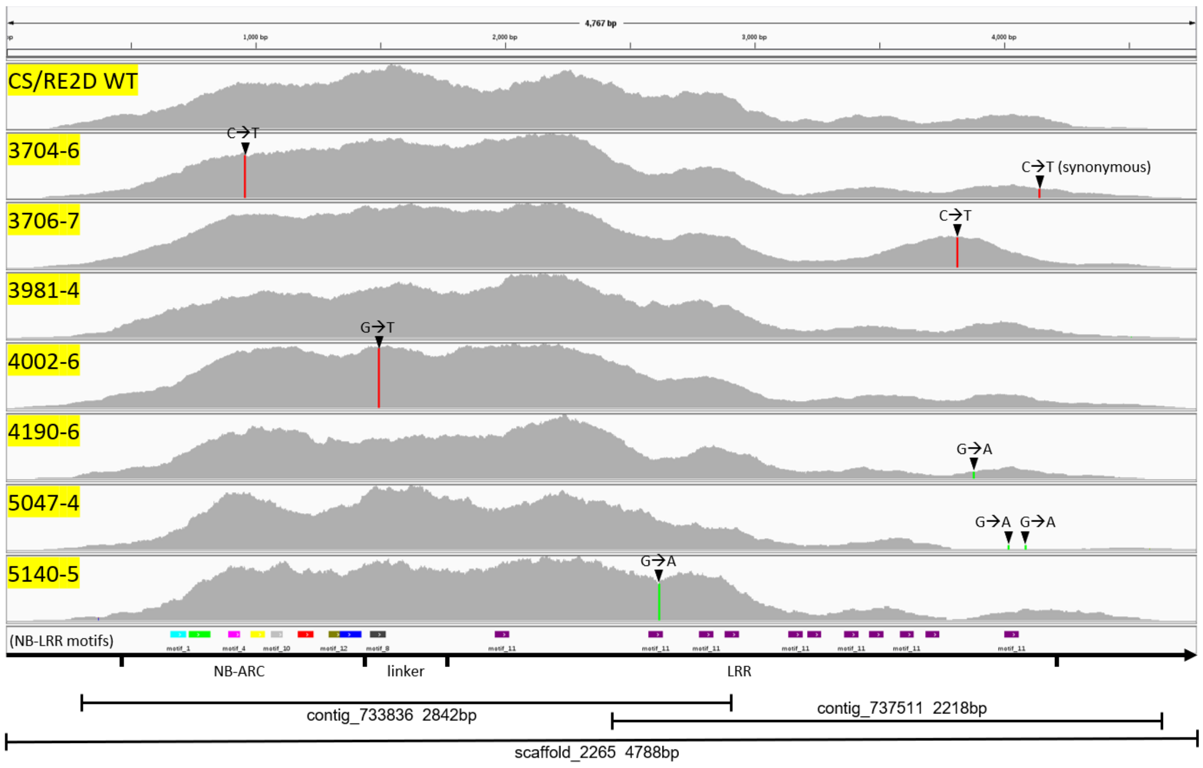

B)

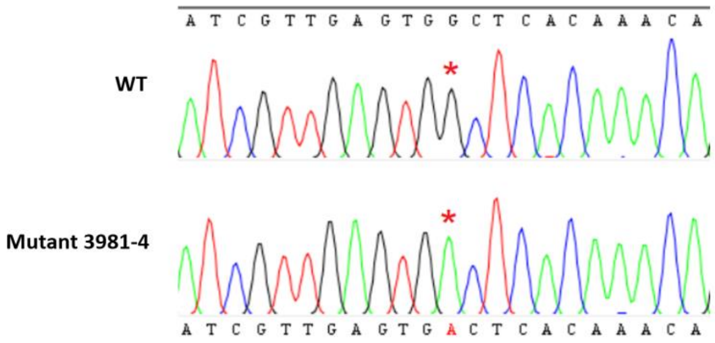

Supplementary Fig. 1. Identification of *Sr6* knockout mutations from mutant sequencing data. A: Histograms of RenSeq read alignments to candidate contigs showing mutation positions. Coverage depth of read alignments to candidate sequences is indicated in grey. Sample IDs are highlighted in yellow. SNPs are denoted with red or green vertical bars that indicate a mismatch with the wildtype (WT) occurring in almost 100% of the overlapping reads. Specific base changes are labelled. Placements of independently assembled contigs are shown. **B:** Region covering a mutation that was not enriched by RenSeq. Position of SNP is indicated by asterisks.

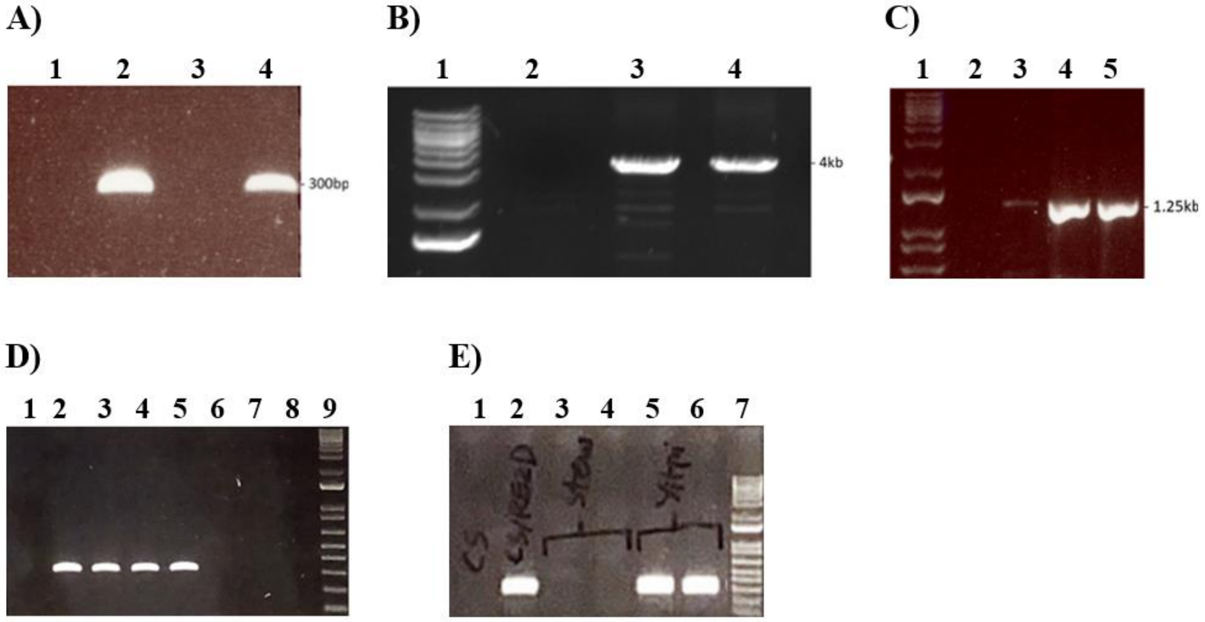

Supplementary Fig. 2. PCR screens from different stages of *Sr6* cloning work. A: Dominant STS marker diagnostic for *Sr6*. *Sr6STS1* was validated in 197 F_3 lines from reciprocal crosses of Chinese Spring and CS/Red Egyptian (RE) 2D substitution line. Agarose gel lanes: 1=CS, 2=CS/RE 2D, 3=Avocet R, 4=Manitou. **B:** PCR product of the main coding region covered by the longest scaffold. Agarose gel lanes: 1=ladder, 2=CS, 3=CS/RE 2D, 4=mutant 5047-4. **C:** PCR product of upstream coding region carrying CC and BED domains. Agarose gel lanes: 1=ladder, 2=water, 3=CS, 4=CS/RE 2D, 5=mutant 3981-4. **D:** Marker *Sr6STS1* on various lines: 1=CS, 2=CS/RE 2D, 3-5=Fielder, 6-8=Westonia, 9=ladder. **E:** Marker *Sr6STS1* on various lines: 1=CS, 2=CS/RE 2D, 3-4=Stewart (durum), 5-6=Yitpi, 7=ladder. Source data are provided as a Source Data file.

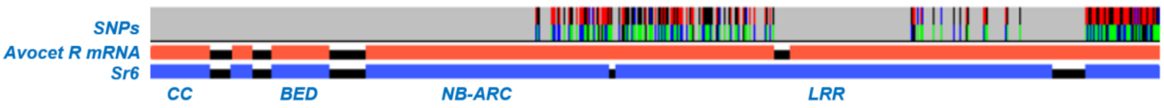

Supplementary Fig. 3. Alignment of *Sr6* and Avocet R transcripts against Landmark reference genome. Transcript of *Sr6* showing structure conservation with the transcript from Avocet R RNAseq. Mismatches with Avocet R are indicated by coloured bars. Gaps in the LRR region of *Sr6* denote extra sequence insertions in Avocet R.

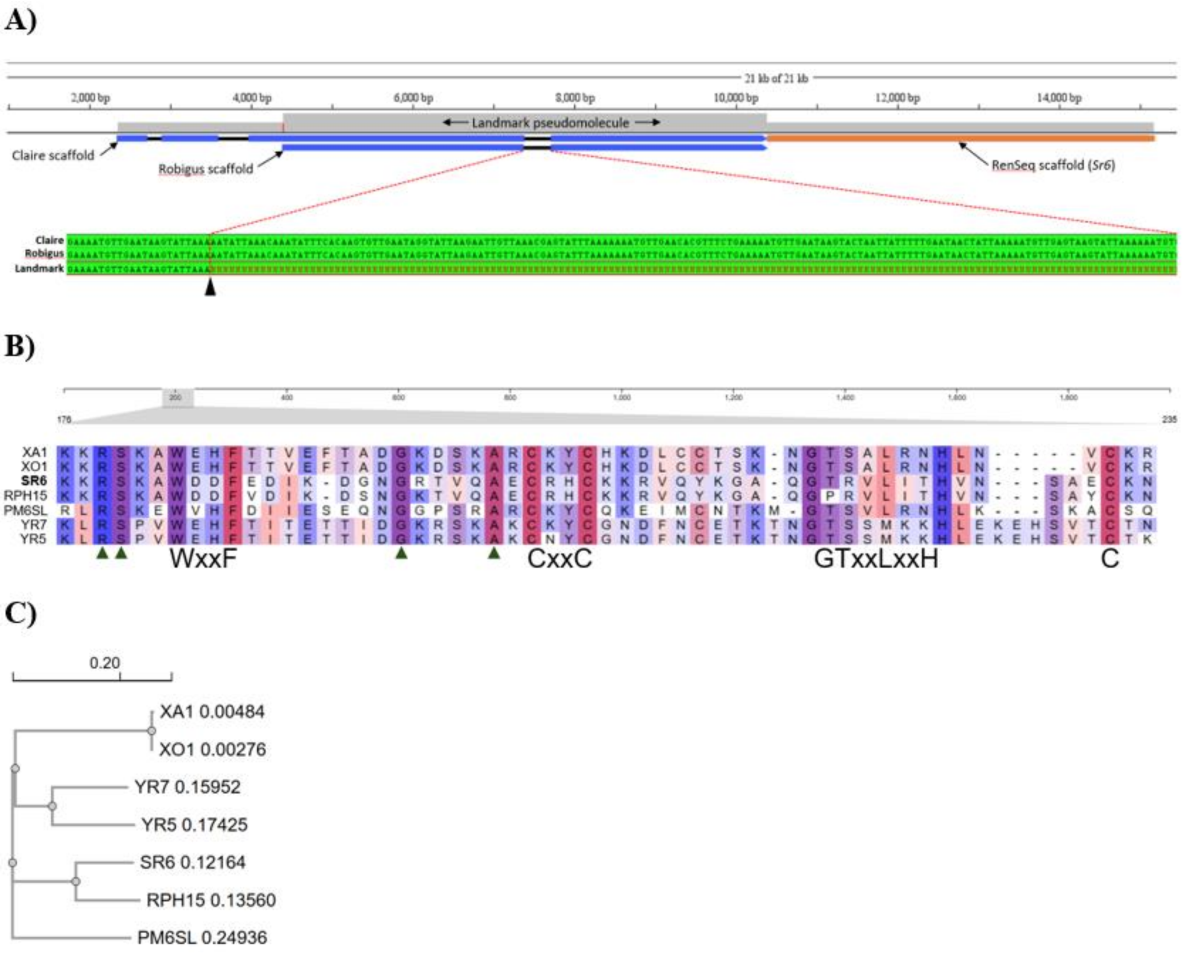

Supplementary Fig. 4. Comparison of *Sr6* sequences with other accessions. A: Gap closing upstream region of *Sr6* by aligning scaffolds from different assemblies. Scaffolds are aligned to Landmark reference genome. The start of the gap interval in Landmark is expanded and indicated by a black arrowhead. **B**: Amino acid sequence alignments of Zf-BED domains from NLRs of *Xa1, Xo1, Sr6*, *Rph15, Pm6Sl, Yr7* and *Yr5*. Motifs that are ~80% conserved in land plants are labelled. Dark green arrowheads indicate additional residues that are conserved across all seven sequences. Sequences are color-coded based on hydrophobicity. **C:** A phylogram based on whole protein sequences of the same seven genes as in (**B**), with branch lengths representing distances.

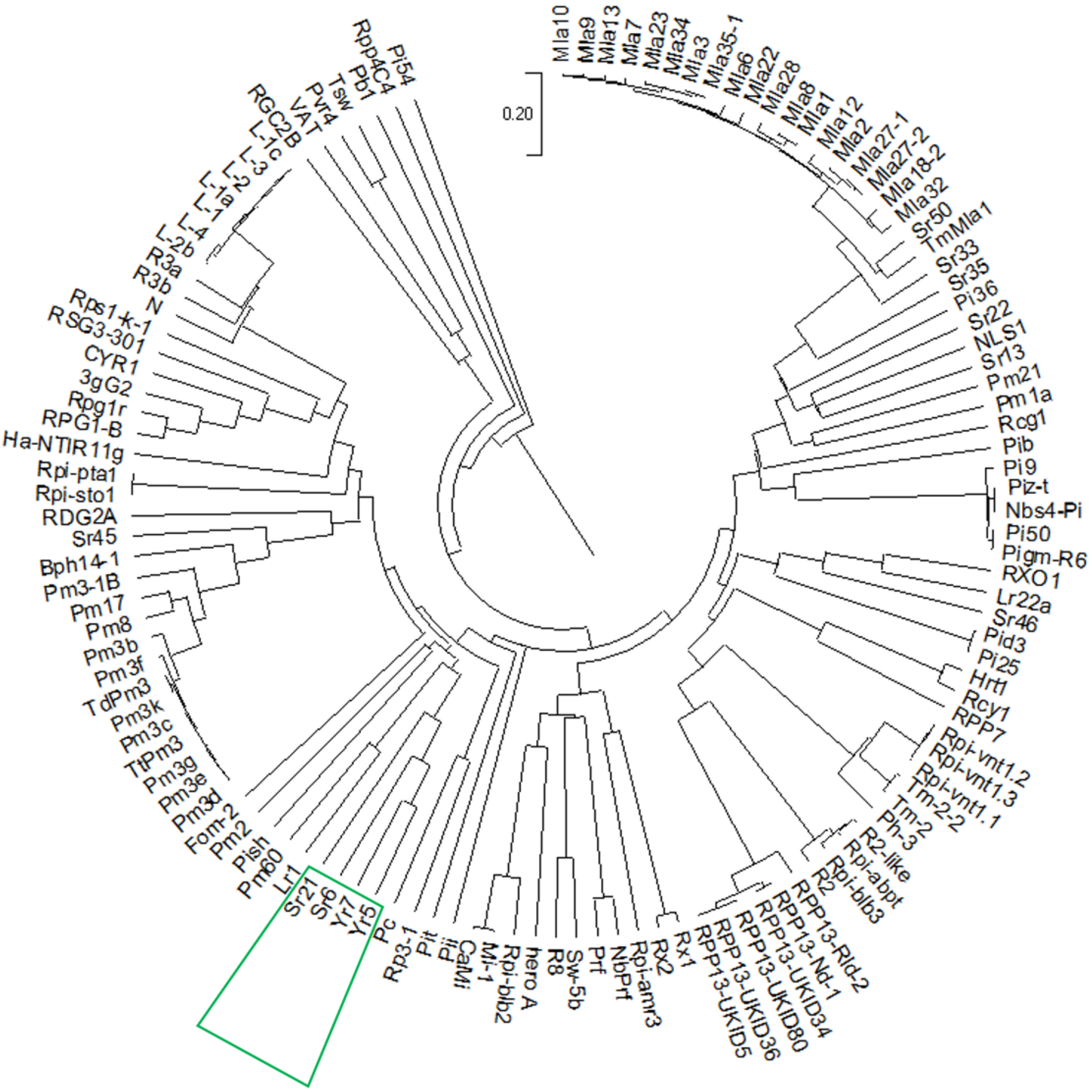

Supplementary Fig. 5. Phylogenetic tree showing *Sr6* among various plant CNL type NB-LRRs. The optimal tree with the sum of branch length = 30.56 is shown. The tree is drawn to scale and the analysis involved 125 amino acid sequences. The group containing *Sr6* is boxed in green.

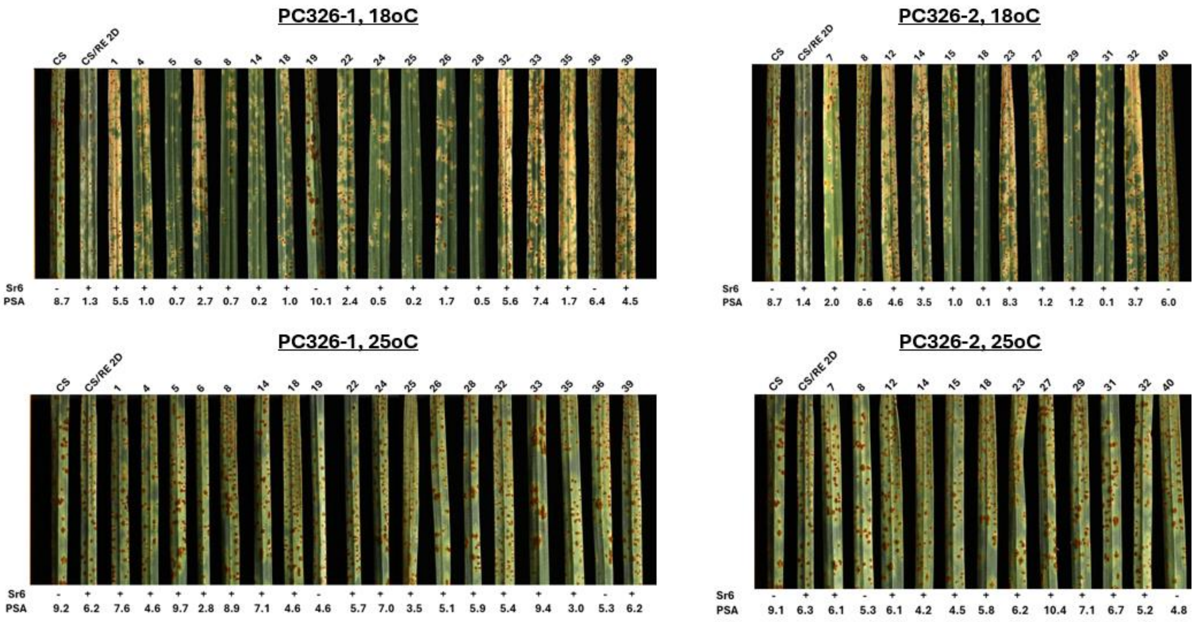

Supplementary Fig. 6. Westonia+*Sr6* transgenic T2 seedling leaves at 14 days post-inoculation with *Pgt* 21-0. All numbered individuals were derived from two independent T_0 parents (PC326-1, PC326-2) and grown at either 18°C (upper panel) or 25°C (lower panel). *Sr6*+ control: CS/RE 2D; *Sr6*- control: CS. Bottom labels indicate the presence/absence (+/-) of *Sr6* as indicated by both the genotypic (Bar gene) and phenotypic data. Images were processed with ASSESS and the percent sporulating area (PSA) is shown under each leaf. Minor leaf rust contamination on the leaves is more evident in the upper panel.

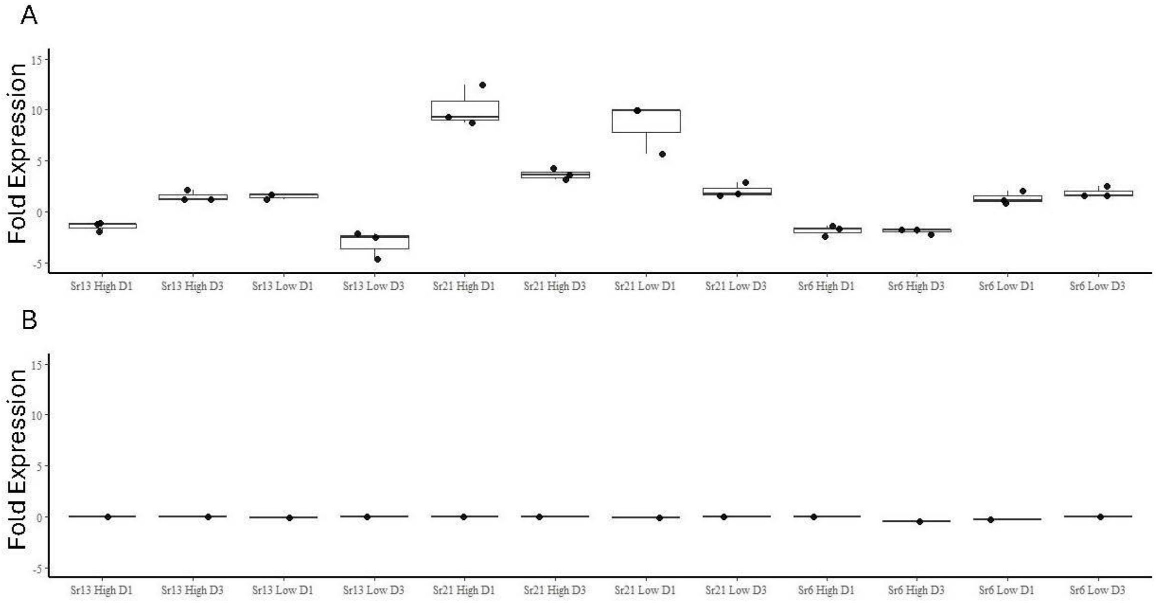

Supplementary Fig. 7. The relative transcript abundance of *Sr6*, *Sr13* and *Sr21* genes in their respective lines. The relative transcript abundance in Sr-LMPG lines was compared with the transcript abundance in LMPG-6. **A**: qPCR analysis of Sr-LMPG lines at various temperature conditions. *Sr6* was slightly upregulated at cold at Day 3 and downregulated at high temperature. *Sr13* was upregulated under low temperature conditions at Day 1 and under high temperature conditions at Day 3. *Sr21* gene was upregulated at all tested temperature conditions and time points. **B**: RNAseq analysis of the *Sr* genes showed no significant expression in rust infected samples under low or high temperature conditions. The scale of the y-axis in both (**A**) and (**B**) is consistent to enable comparison. For qPCR analyses, relative transcript abundance was considered statistically significant with log fold change ± 1.5 and P value of <0.05. Boxplots denote median values with second and third quartiles and with whiskers extending to the most extreme values, but no further than 1.5 times the inter-quartile range. Source data are provided as a Source Data file.

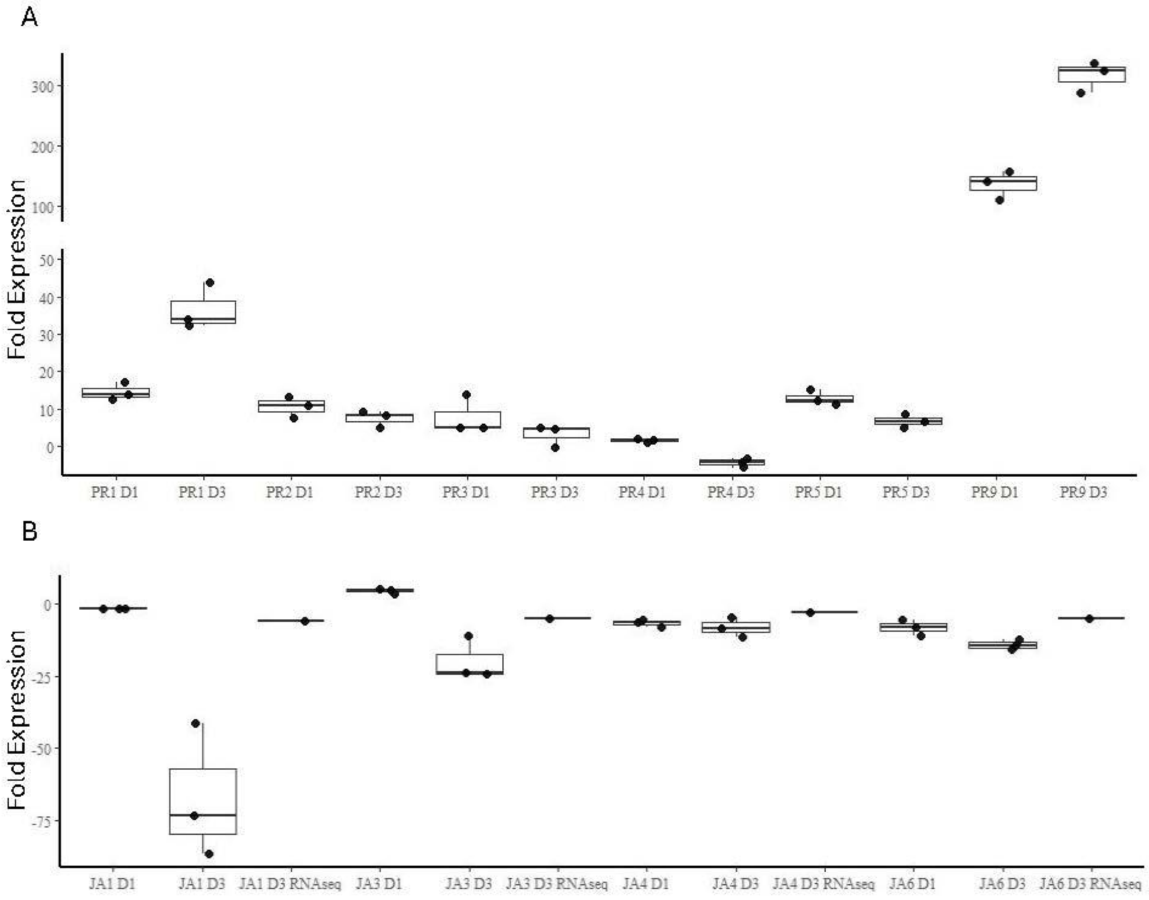

Supplementary Fig. 8. qPCR validation of pathogenesis related (PR) genes (*PR1*, *PR2*, *PR3*, *PR4*, *PR5* and *PR9*) and JA genes (*JA1*, *JA3*, *JA4* and *JA6*) that are expressed in *Pgt* infected *Sr6* lines at low temperature. Expression of PR (A) and JA (B) genes was validated with qPCR. The expression of PR genes followed similar expression patterns at Day 1 and Day 3 and almost all the tested candidates were upregulated. From the four tested JA genes, JA3 was upregulated at Day 1 but all other genes were downregulated. At Day 3 all four JA genes were downregulated (RNAseq) and validated with qPCR. The JA genes mostly follow the same expression pattern and were downregulated. For qPCR analyses, relative transcript abundance was considered statistically significant with log fold change ± 1.5 and *P* value of <0.05. Boxplots denote median values with second and third quartiles and with whiskers extending to the most extreme values, but no further than 1.5 times the inter-quartile range. Source data are provided as a Source Data file.

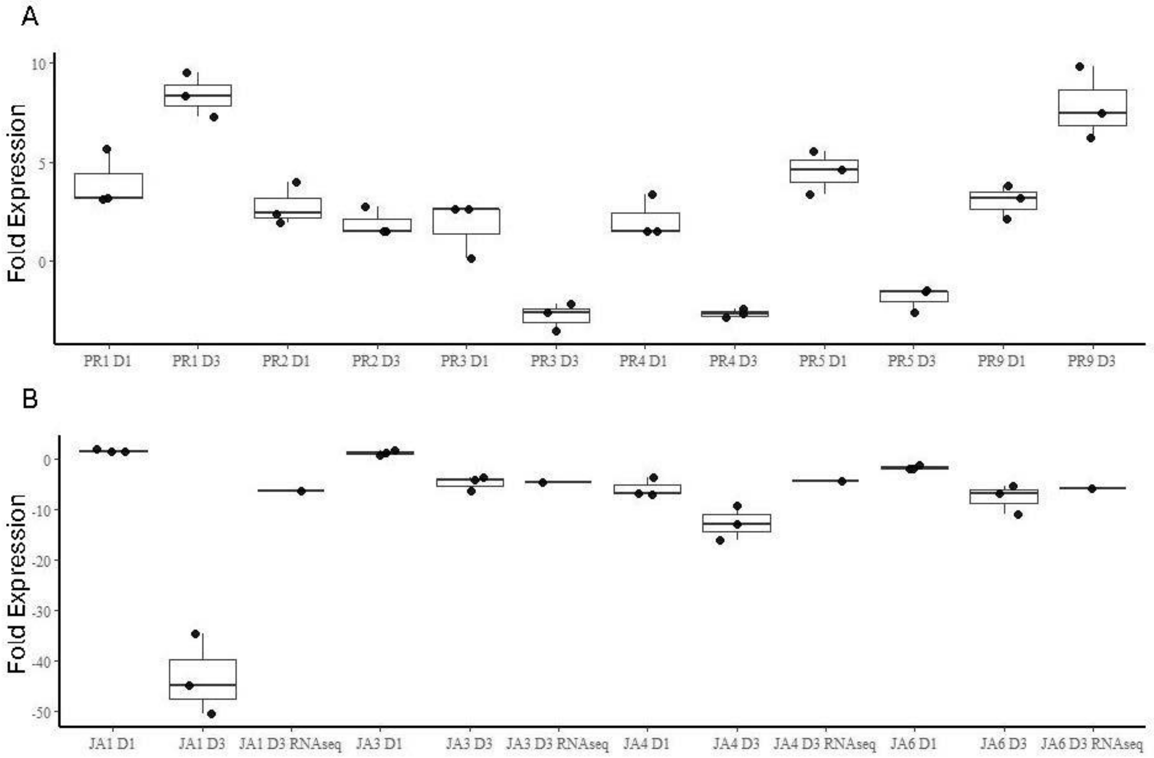

Supplementary Fig. 9. Relative expression of the PR and JA genes expressed in *Pgt*-infected lines with *Sr13*. Gene expression was validated with qPCR for Day 1 and Day 3 relative to transcript abundance in LMPG. **A:** All six tested PR genes (*PR1*, *PR2*, *PR3*, *PR4*, *PR5*, *PR9*) were upregulated at Day 1 whereas *PR3*, *PR4* and *PR5* were downregulated at Day 3. **B:** JA genes, *JA1* and *JA3* were upregulated at Day 1. All JA genes were downregulated at Day 3 [RNAseq and qPCR]. For qPCR analyses, relative transcript abundance was considered statistically significant with log fold change ± 1.5 and *P* value of <0.05. Boxplots denote median values with second and third quartiles and with whiskers extending to the most extreme values, but no further than 1.5 times the inter-quartile range. Source data are provided as a Source Data file.

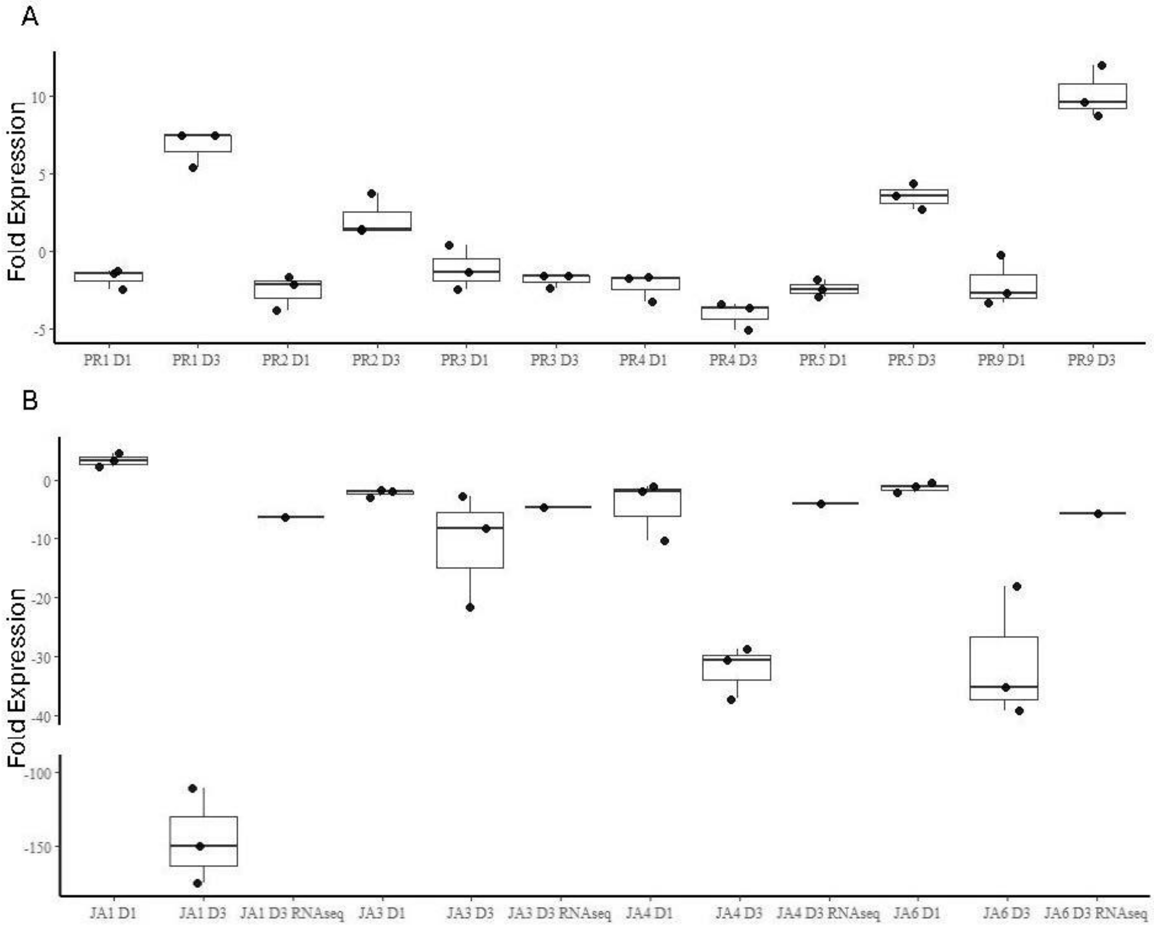

Supplementary Fig. 10. Fold expression of PR (*PR1*, *PR2*, *PR3*, *PR4*, *PR5* and *PR9*) and JA (*JA1*, *JA3*, *JA4* and *JA6*) genes expressed in *Pgt* infected lines with *Sr21* at Day 1 and Day 3. The expression of PR (A) and JA (B) genes were validated with qPCR. All PR genes except *PR3* and *PR4* were upregulated at Day 1, but all were downregulated at Day 3. JA genes were downregulated at both Day 1 and Day 3 except *JA1* which was upregulated at Day 1. For qPCR analyses, relative transcript abundance was considered statistically significant with log fold change ± 1.5 and *P* value of <0.05. Boxplots denote median values with second and third quartiles and with whiskers extending to the most extreme values, but no further than 1.5 times the inter-quartile range. Source data are provided as a Source Data file.

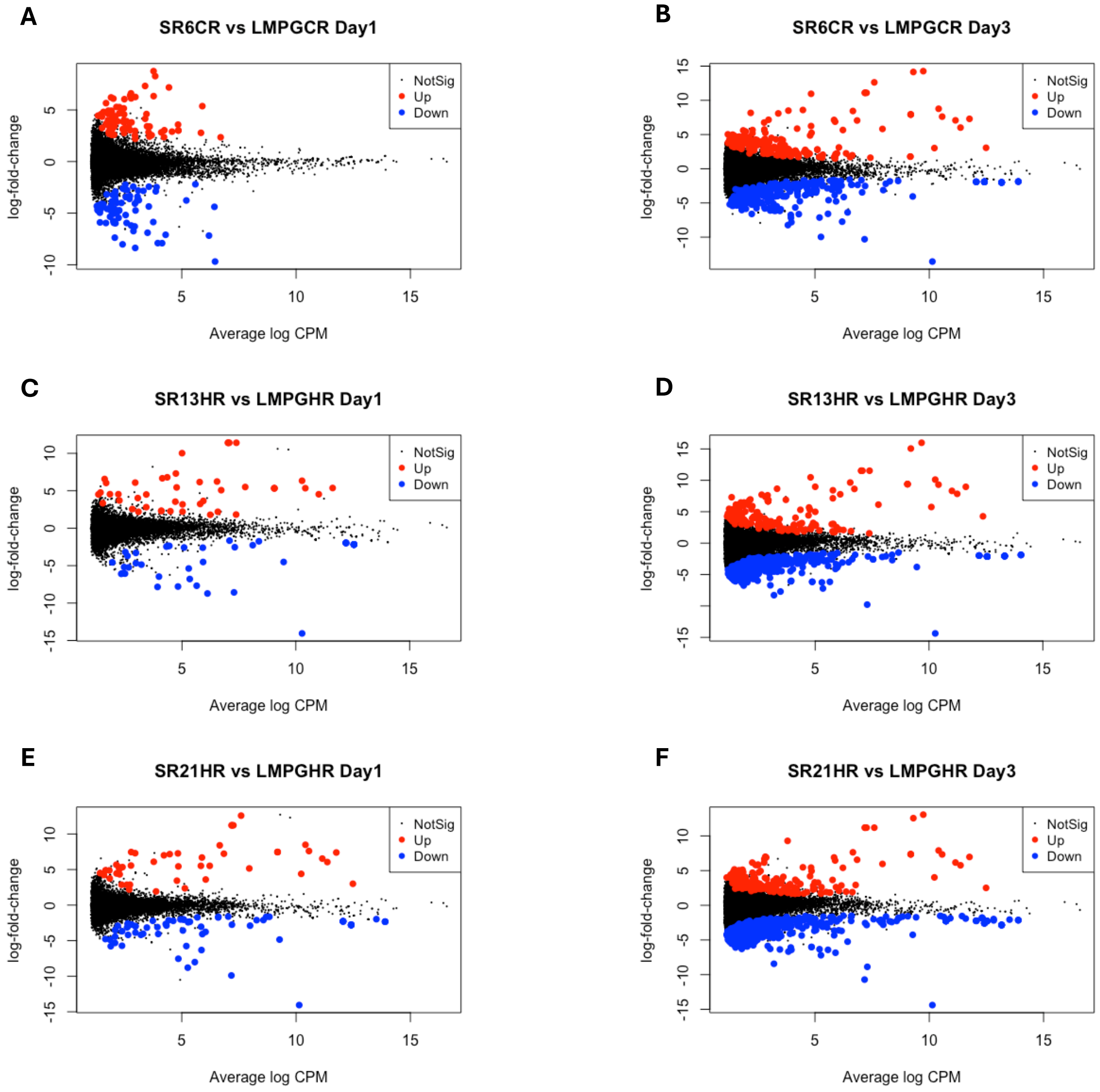

Supplementary Fig. 11. Volcano plot distribution of differential gene expression in LMPG near-isogenic lines compared to LMPG. A: LMPG-*Sr6* at low temperature at Day 1. **B:** LMPG-*Sr6* at low temperature at Day 3. **C:** LMPG-*Sr13* at high temperature at Day 1. **D:** LMPG-*Sr13* at high temperature at Day 3. **E:** LMPG-*Sr21* at high temperature at Day 1. **F:** LMPG-*Sr21* at high temperature at Day 3. The significantly differentially expressed genes (log fold change >= 2 and FDR (false discovery rate) of <0.05) are represented by red (upregulated) or blue (downregulated) dots. Black dots represent non-significant genes. X axis represents log fold change and Y axis represents average counts per million.

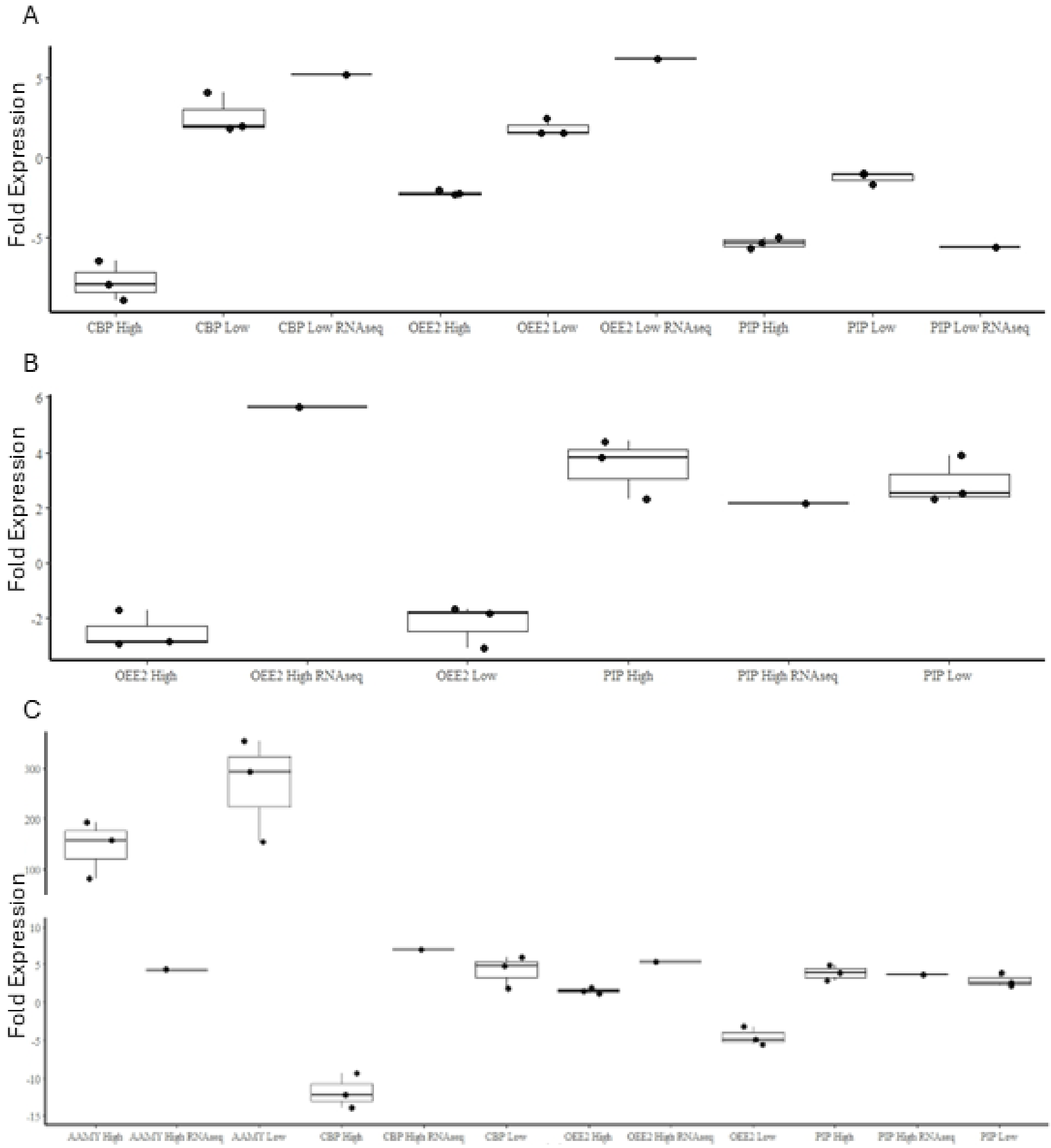

Supplementary Fig. 12. Confirmation of the relative transcript abundance in *Pgt* infected *Sr6*, *Sr13* and *Sr21* lines at Day 3 using RNAseq and qPCR analyses. Gene validation was performed using qPCR and RNAseq analyses. The relative transcript abundance in Sr-LMPG lines was compared with the transcript abundance in LMPG-6. **A**: SR6 vs LMPG Day 3. OEE-2 and CBP were upregulated at low temperature but downregulated at high temperature. The PIP1 was downregulated at both high and low temperatures. qPCR values were compared to RNAseq data (fold expression) at low temperature. OEE-2 and CBP were upregulated, and PIP1 was downregulated in RNAseq analysis. **B**: SR13 vs LMPG Day 3. In RNAseq, PIP1 and OEE-2 were upregulated at both low and high temperatures. qPCR analysis showed PIP1 was upregulated and OEE-2 was downregulated at both temperatures. **C**: SR21 vs LMPG Day 3. RNAseq analysis showed all four selected genes were upregulated. qPCR analysis showed PIP1 and AAMY were upregulated and OEE-2 and CBP were downregulated at both low and high temperature, respectively. AAMY was significantly upregulated at all tested temperature conditions. For qPCR analyses, relative transcript abundance was considered statistically significant with log fold change ± 1.5 and *P* value of <0.05. Boxplots denote median values with second and third quartiles and with whiskers extending to the most extreme values, but no further than 1.5 times the inter-quartile range. Source data are provided as a Source Data file.

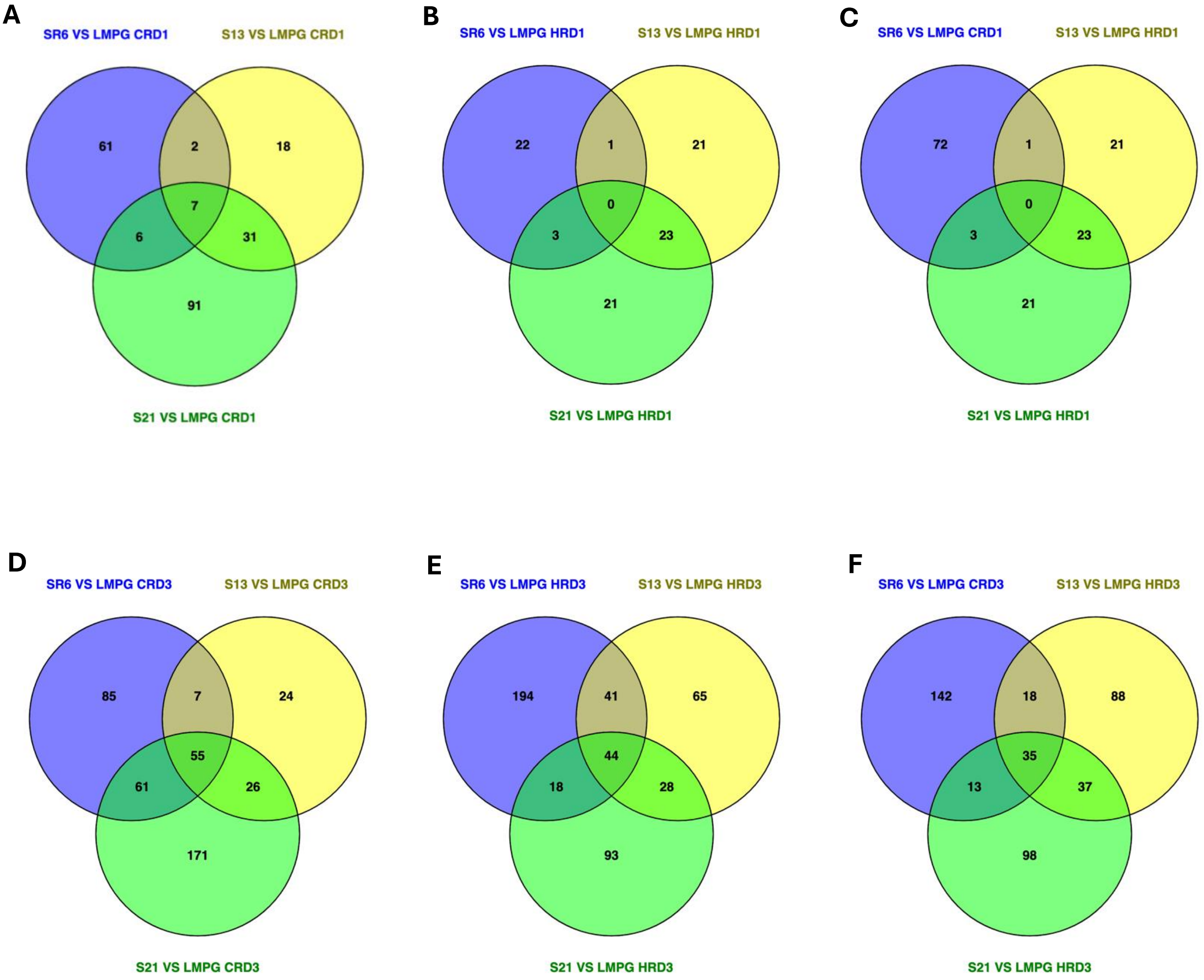

Supplementary Fig. 13. Venn Diagrams indicating the number of upregulated genes in LMPG-*Sr6*, LMPG-*Sr13*, and LMPG-*Sr21* relative to LMPG at **A:** low temperature at Day 1, **B:** high temperature at Day 1, **C:** temperatures at which each temperature-sensitive gene is most effective at Day 1, **D:** low temperature at Day 3, **E:** high temperature at Day 3, and **F:** temperatures at which each temperature-sensitive gene is most effective at Day 3. Source data are provided as a Source Data file.

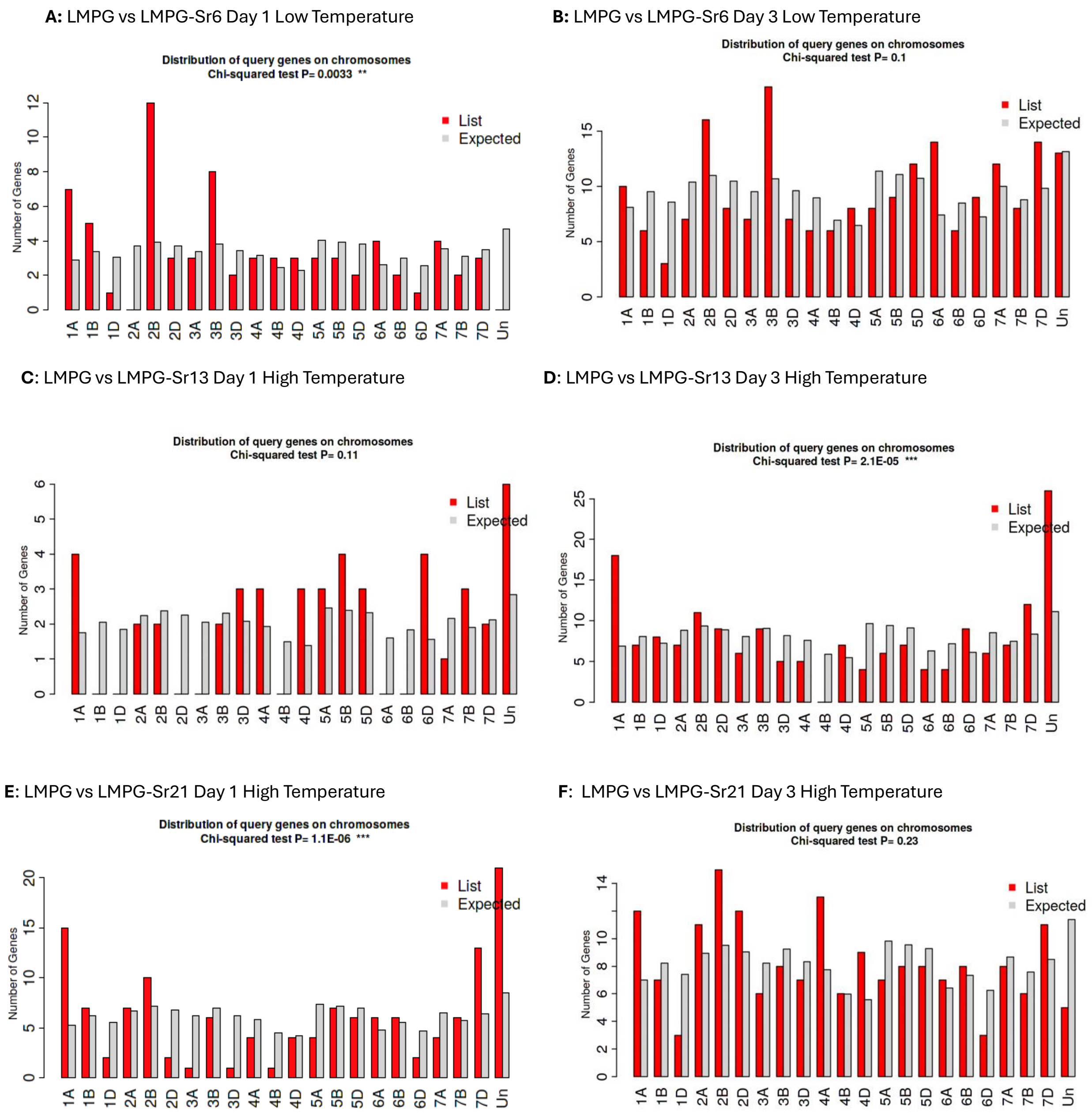

Supplementary Fig. 14. Distribution of genes upregulated in *Sr*-containing lines and downregulated in LMPG across wheat chromosomes. Genes upregulated in line LMPG-*Sr6* under low temperature on Day 1 (**A**) and Day 3 (**B**). Genes upregulated in LMPG-*Sr13* under high temperature on Day 1 (**C**) and Day 3 (**D**). Genes upregulated in LMPG-*Sr21* under high temperature on Day 1 (**E**) and Day 3 (**F**). Red color indicates number of DEGs that mapped to each chromosome and the grey color is the expected distribution of the genes across each chromosome. The Y axis indicates the number of genes whereas the x axis shows chromosome names including unmapped reads at the end. Source data are provided as a Source Data file.

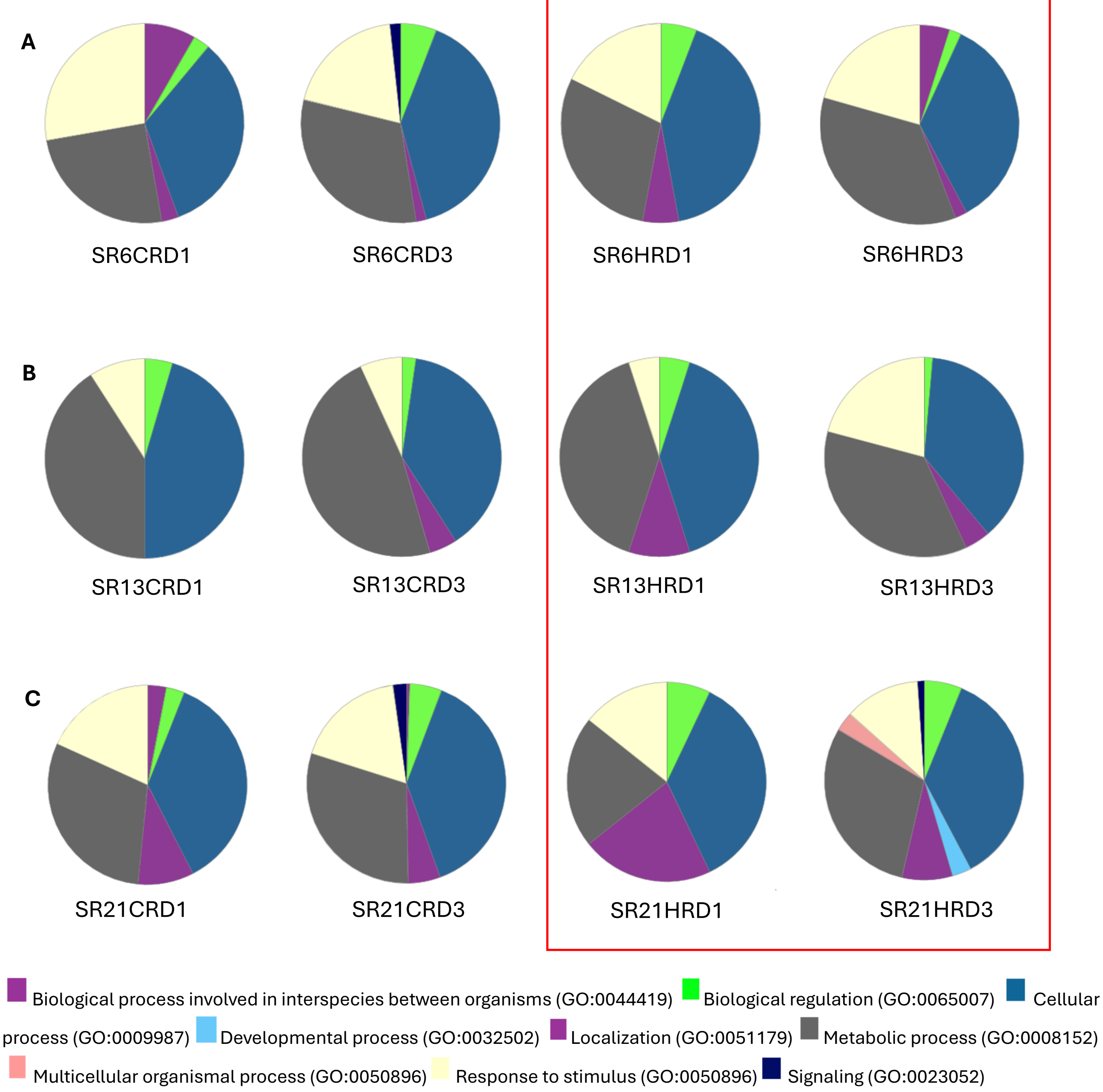

Supplementary Fig. 15. Pie chart distribution of differentially expressed genes in lines with *Sr* genes. A: *Sr6*; About one fourth of genes in the *Sr6* line were associated with response to stimulus (yellow). Genes associated with signalling (dark blue) were seen only under low temperature at Day 3. Number of genes associated with biological processes (light green) were higher at Day 3 under low temperature but low under high temperature conditions. Genes with interspecies interaction (purple) are seen at Day 1 at low temperature and at Day 3 under high temperature. Genes associated to metabolic processes are higher at Day 3 under both temperature conditions. A significant number of genes involving in cellular processes (blue) were differentially expressed in all conditions. Localization genes (purple 2nd) were higher at Day 1 and were gradually reduced at Day 3 in all conditions. **B:** *Sr13*; Response to stimulus genes (yellow) were higher at Day 1 but slightly reduced at Day 3 under low temperature conditions. Under high temperature these genes were drastically increased at Day 3. Biological regulation (light green) genes were reduced at Day 3 at both temperature conditions. Cellular (blue) and metabolic (gray) process genes were higher under low temperature conditions. Localization (purple 2nd) genes missing under low temperature were expressed under high temperature at Day 1; a similar number of genes are expressed at Day 3 at both conditions.
C: *Sr21*; Response to stimulus genes (yellow) were slightly higher under low temperature conditions. Genes associated with signalling (dark blue) were observed only at Day 3 and the

number was higher under low temperature. Genes associated with biological processes involved in interspecies between organisms (purple) were seen only under low temperature and the number was higher at Day 1. Biological regulation genes (light green) were observed under all conditions and the number was slightly higher under high temperature. The number of localization (purple 2nd) genes were higher at Day 1 and lower at Day 3 under both temperature conditions but higher at the high temperature compared to low temperature conditions. One-third of the cellular process genes (blue) were expressed under all conditions. The number of metabolic process (grey) genes were almost similar under low temperature but higher at Day 3 under high temperature. Developmental process (light blue) and multicellular organismal process (pink) genes were observed only at Day 3 under high temperature conditions. CR=Low temp, rust inoculated; HR=High temp, rust inoculated, D1=Day 1, D3=Day 3. Each color represents genes associated with cellular functions or pathways. Charts inside the red box correspond to the high temperature condition and outside the red box correspond to the low temperature condition. Source data are provided as a Source Data file.

A: SR6CRD1 VS LMPGCRD1

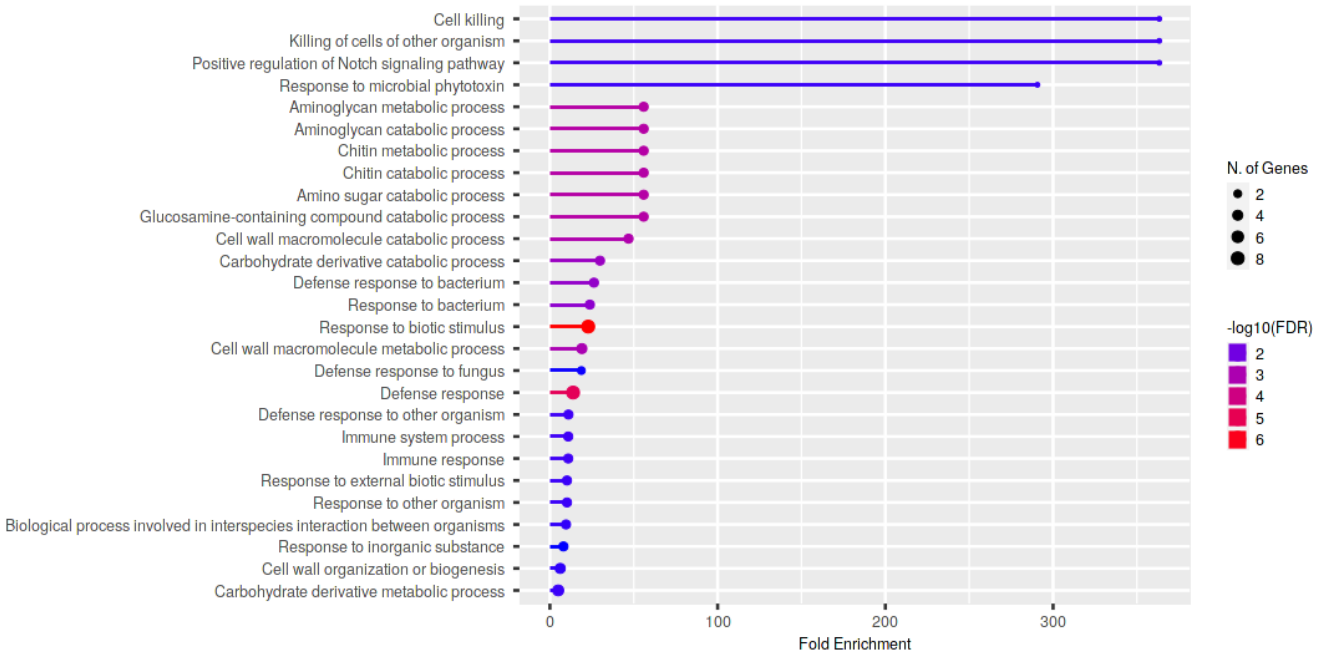

B: SR6CRD3 VS LMPGCRD3

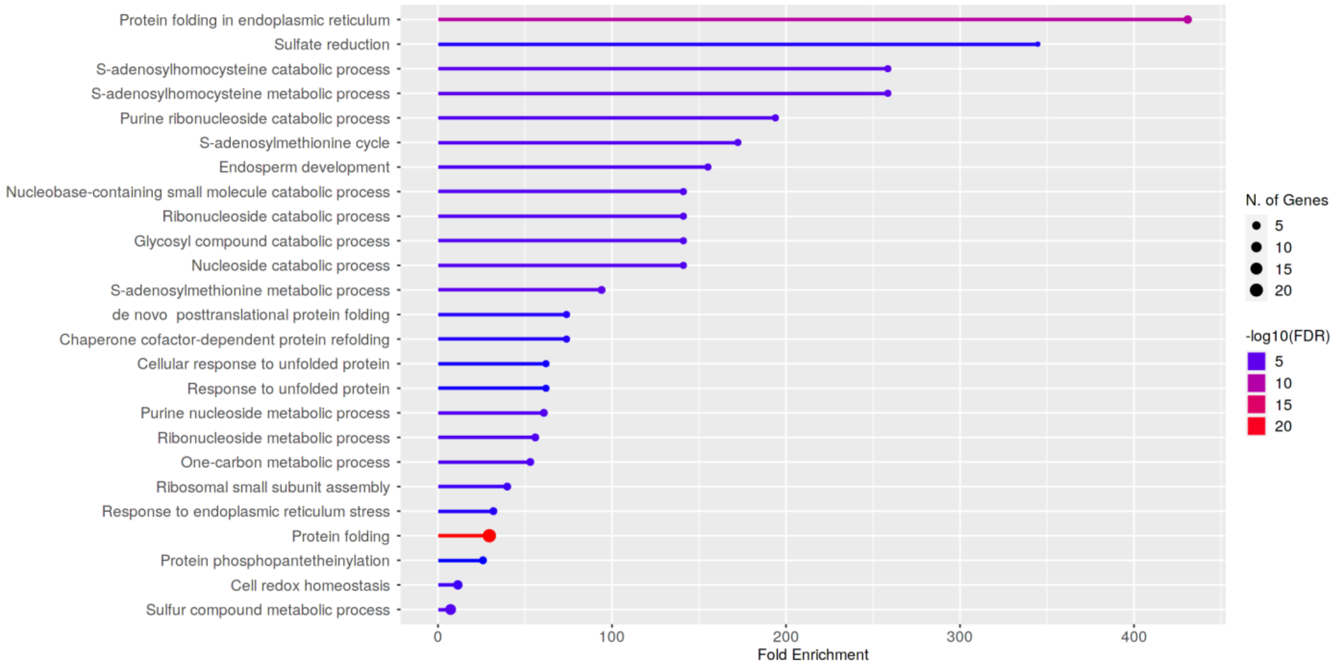

Supplementary Fig. 16. Fold enrichment analysis of genes upregulated in LMPG+*Sr6* under low temperature. A: Genes/pathways expressed at Day 1, **B:** Genes/pathways expressed at Day 3. Round dots represent the number of genes, where larger size represents a higher number of genes. X axis represents the fold enrichment calculated based on the number of genes associated with the pathways/biological processes. Y axis represents pathway. Red color represents the most significant process and blue is less significant in terms of -log10(FDR).

A: SR6HRD1 VS LMPGHRD1

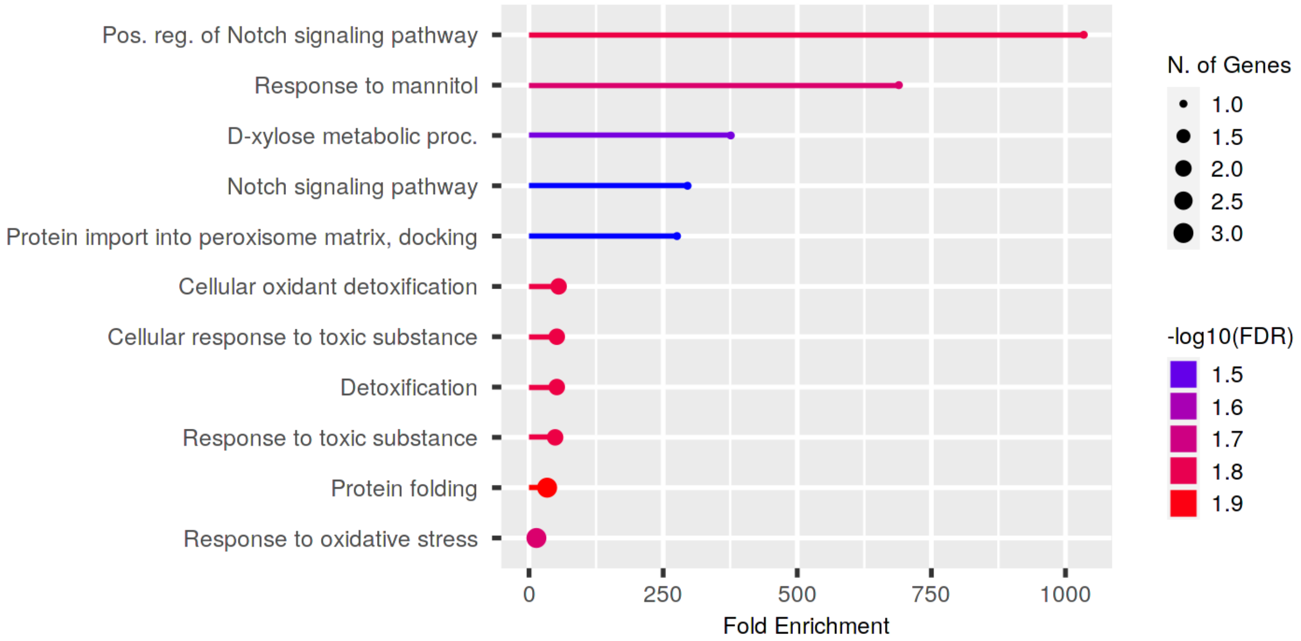

B: SR6HRD3 VS LMPGHRD3

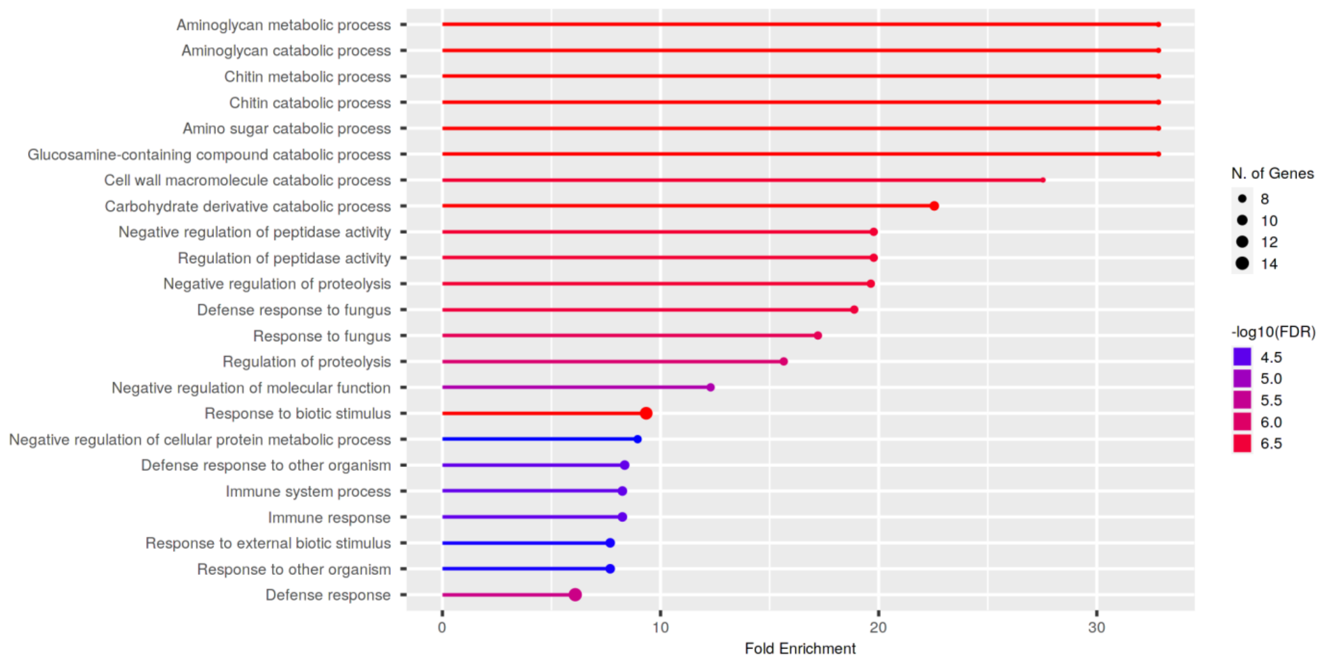

Supplementary Fig. 17. Fold enrichment analysis of genes upregulated in LMPG+*Sr6* under high temperature. A: Genes/pathways expressed at Day 1, **B:** Genes/pathways expressed at Day 3. Round dots represent the number of genes where larger dots represent a higher number of genes. X axis represents the fold enrichment calculated based on the number of genes associated with the pathways/biological process. Y axis represents pathway. Red color represents the most significant process and blue is the less significant in terms of -log10(FDR).

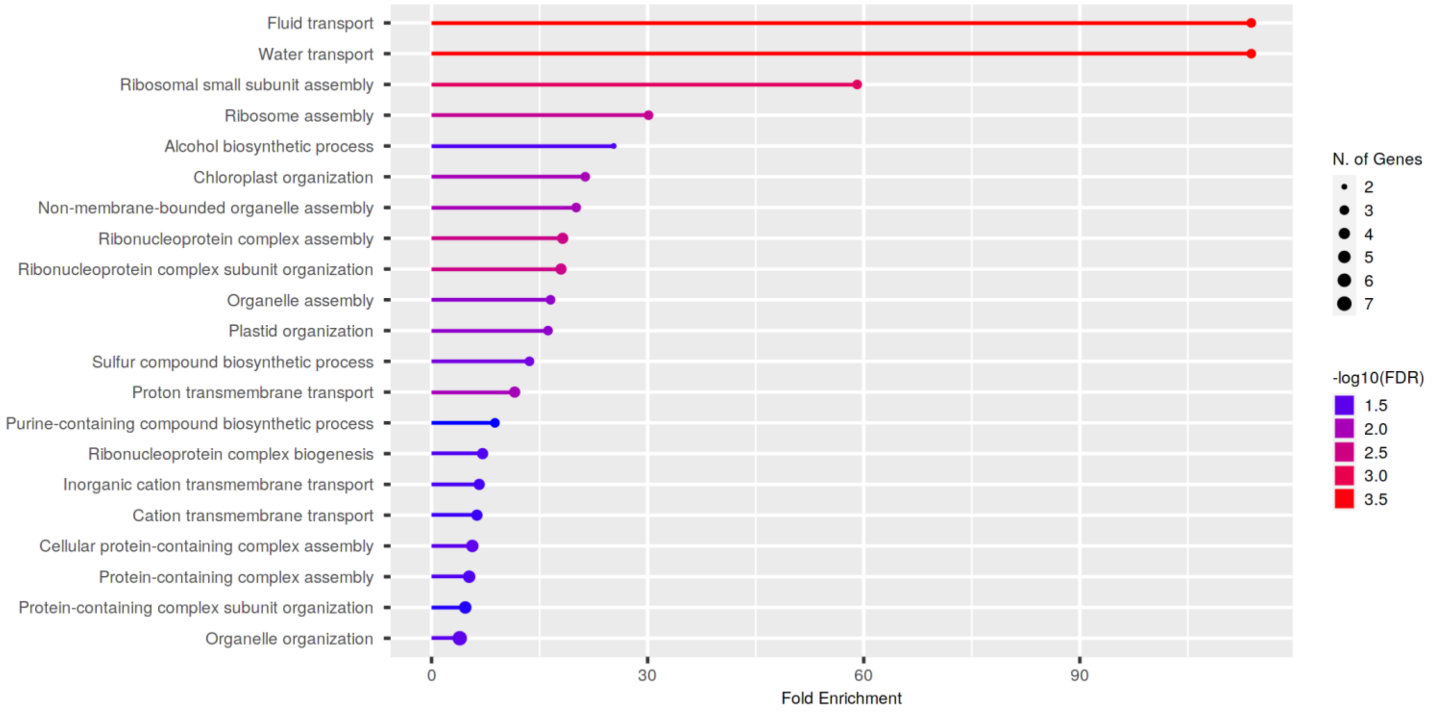

SR13CRD3 VS LMPGCRD3

Supplementary Fig. 18. Fold enrichment analysis of genes upregulated in LMPG+*Sr13* under low temperature. Round dots represent the number of genes with larger dots representing a higher number of genes. X axis represents the fold enrichment calculated based on the number of genes associated with particular pathways/biological process. Y axis represents pathway. Red color represents the most significant processes and blue is the less significant processes in terms of -log10(FDR). No significant pathway was observed at Day 1, low temp.

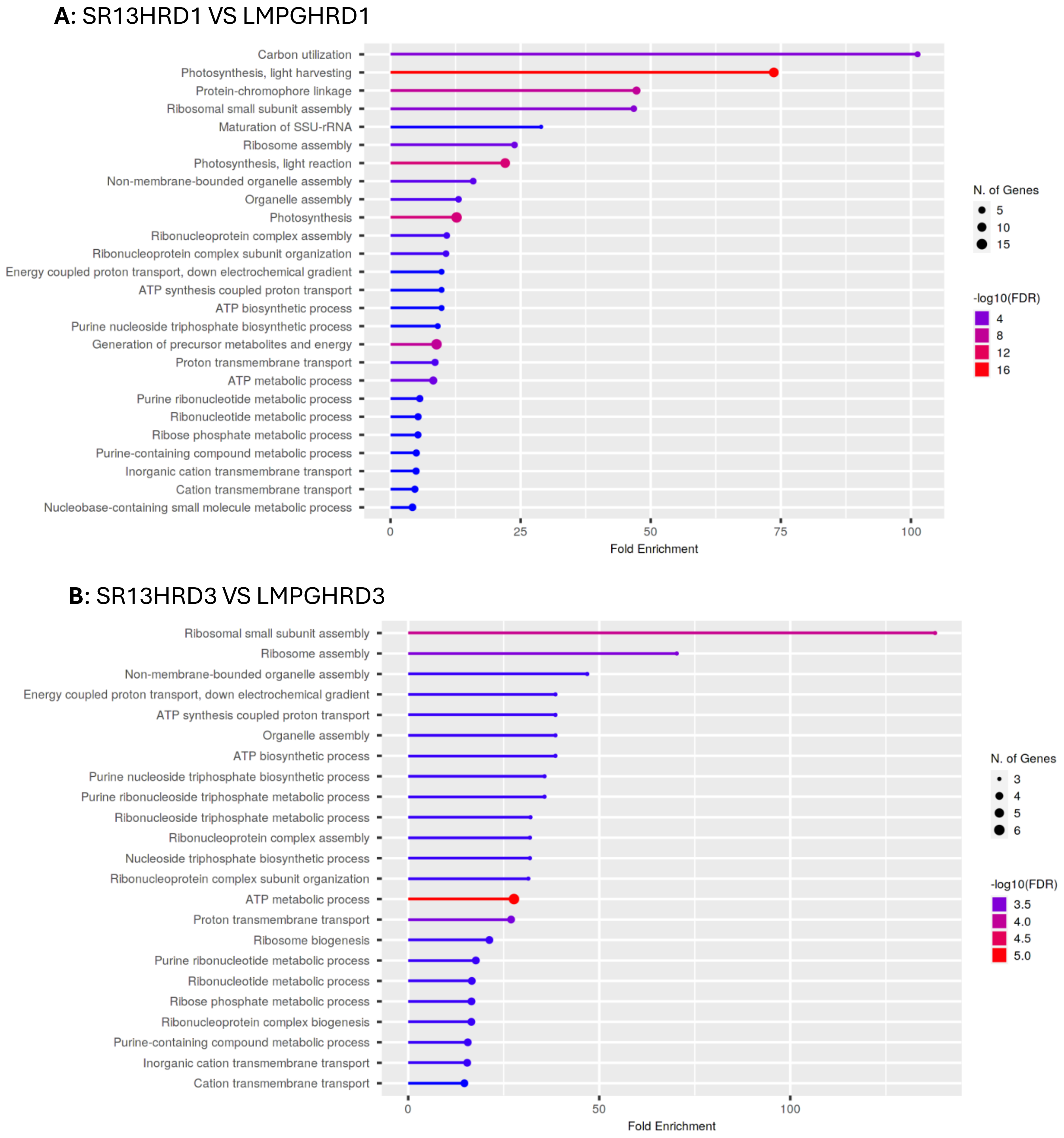

Supplementary Fig. 19. Fold enrichment analysis of genes upregulated in LMPG+*Sr13* under high temperature. **A:** Genes/pathways expressed at Day 1, **B:** Genes/pathways expressed at Day 3. Round dots represent the number of genes where larger dots represent a higher number of genes. X axis represents the fold enrichment calculated based on the number of genes associated with the particular pathway/biological process. Y axis represents the pathway. Red color represents the most significant process and blue are less significant in terms of -log10(FDR).

A: SR21CRD1 VS LMPGCRD1

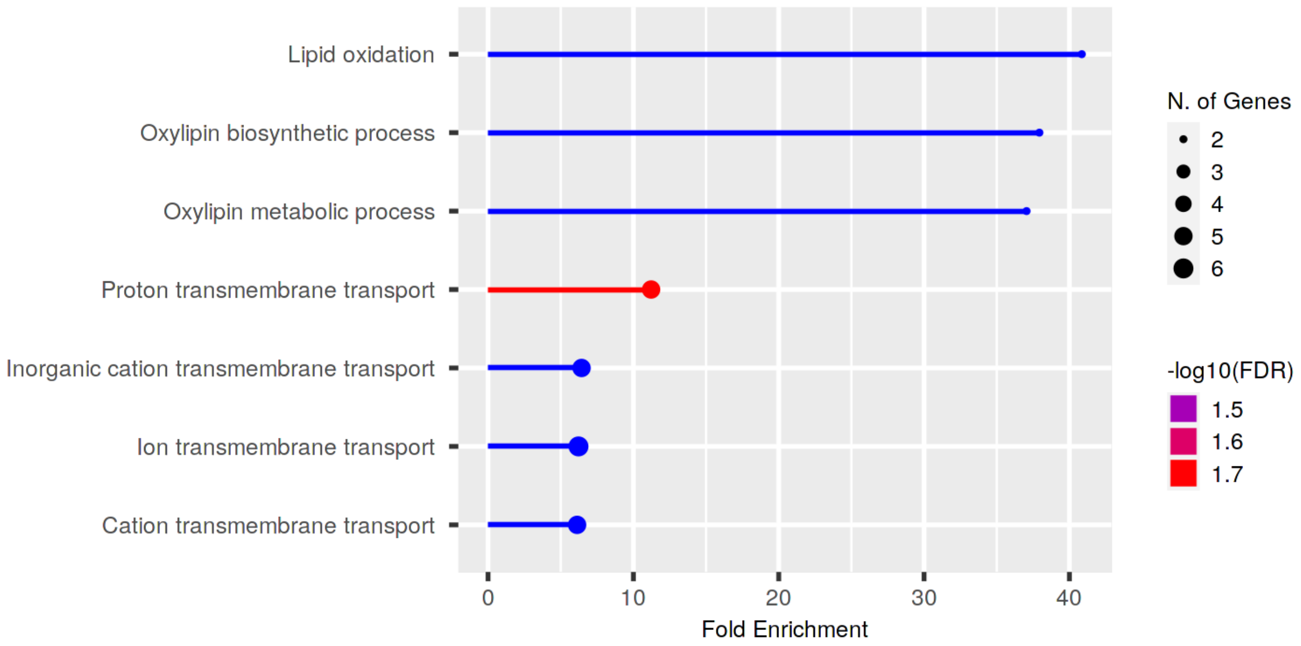

B: SR21CRD3 VS LMPGCRD3

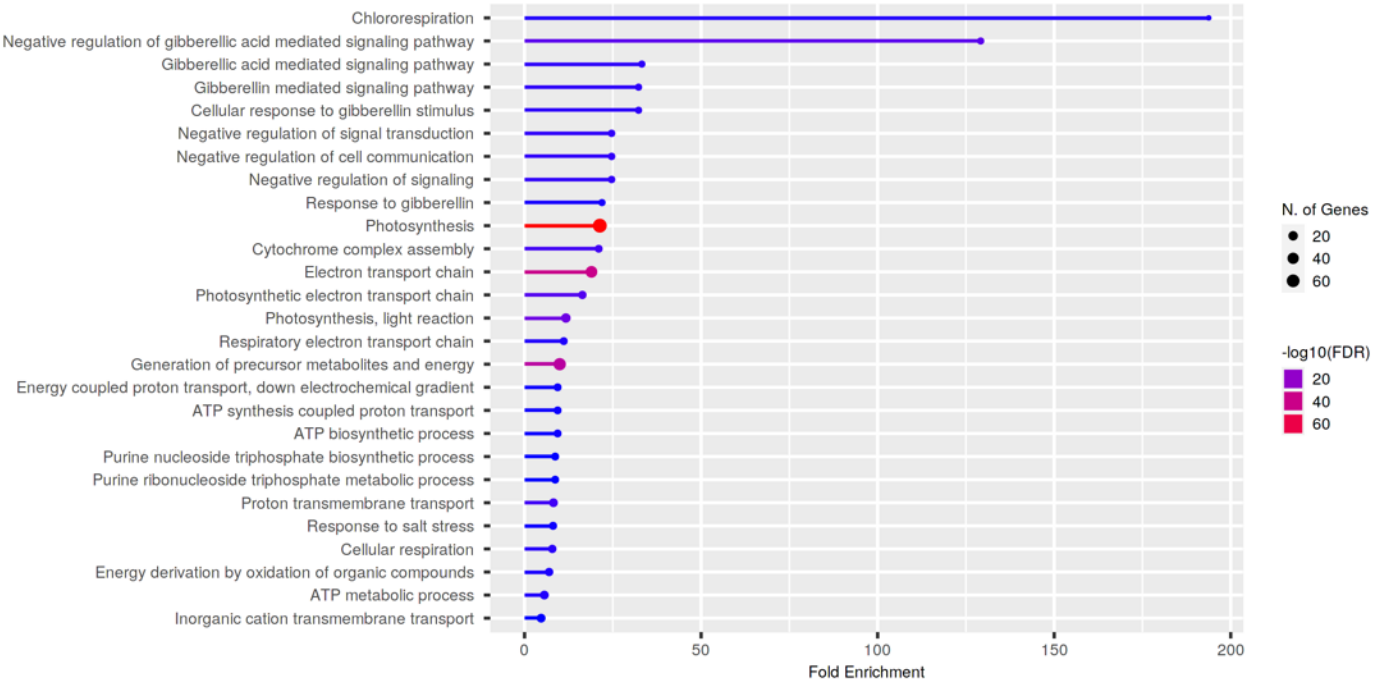

Supplementary Fig. 20. Fold enrichment analysis of genes upregulated in LMPG+*Sr21* under low temperature. A: Genes/pathways expressed at Day 1; **B:** Genes/pathways expressed at Day 3. Round dots represent the number of genes where larger dots represent higher number of genes. X axis represents the fold enrichment calculated based on the number of genes associated to the pathway/biological process. Y axis represents pathway. Red color represents the most significant process and blue are less significant in terms of -log10(FDR).

A: SR21HRD1 VS LMPGHRD1

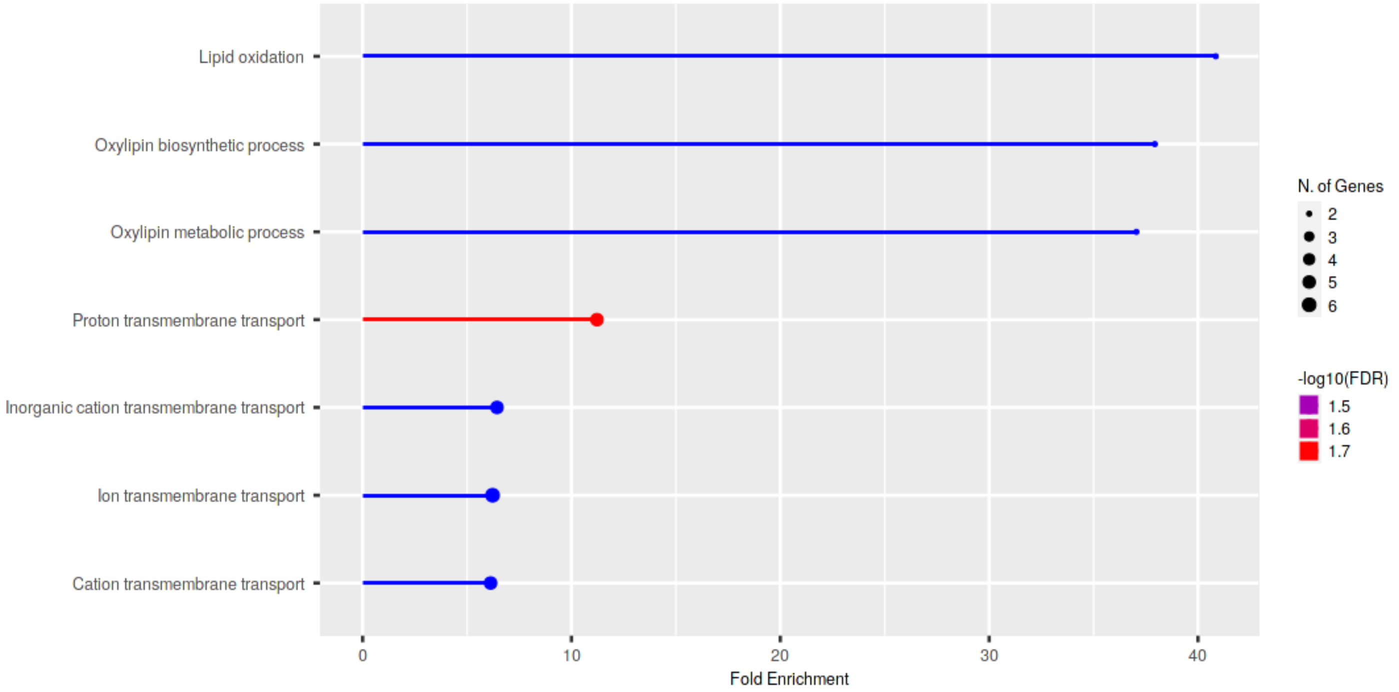

B: SR21HRD3 VS LMPGHRD3

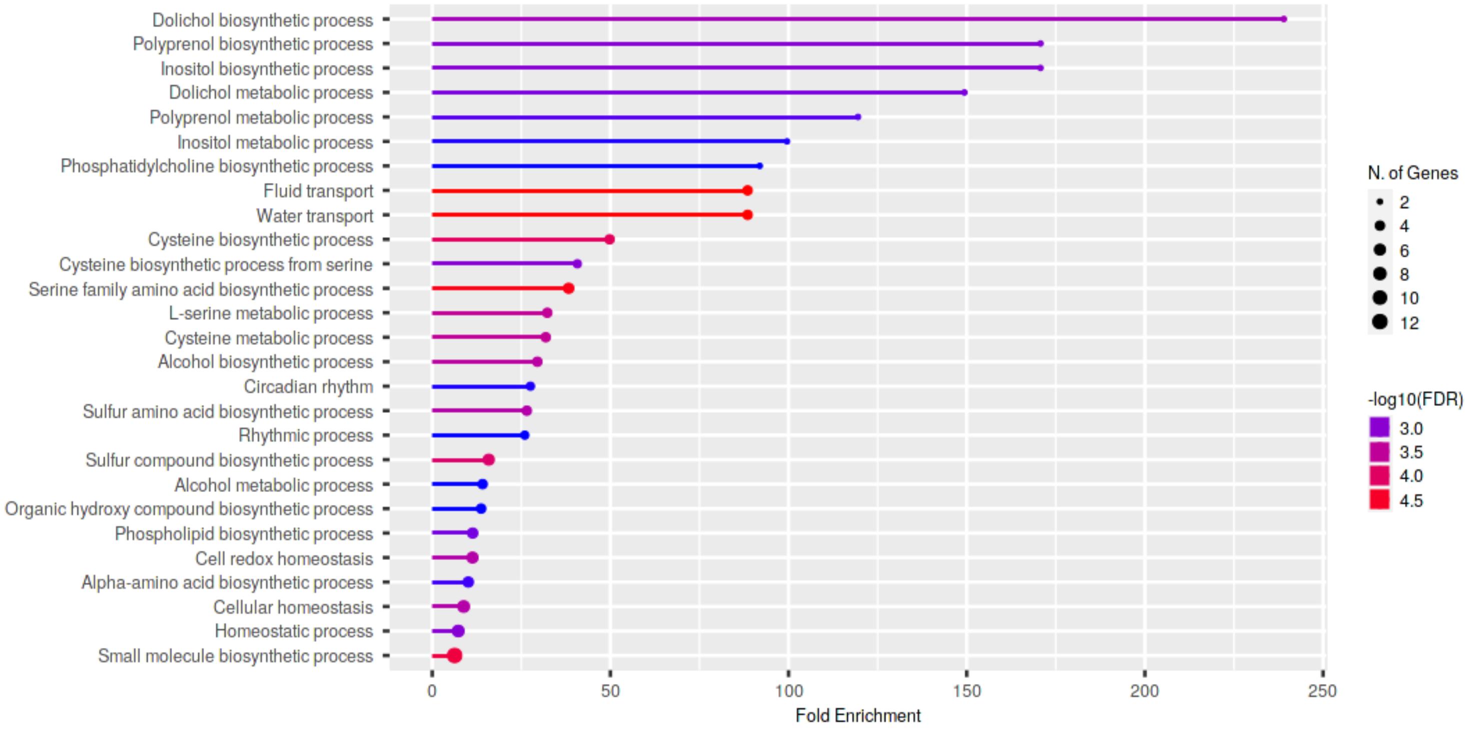

Supplementary Fig. 21. **Fold enrichment analysis of genes upregulated in LMPG+*Sr21* under high temperature. A:** Genes/pathways expressed at Day 1; **B:** Genes/pathways expressed at Day 3. Round or bubble dot represents the number of genes where larger dots represent higher number of genes. X axis represents the fold enrichment calculated based on the number of genes associated with the particular pathway/biological process. Y axis represents pathway. Red color represents the most significant process and blue represents the less significant in terms of -log10(FDR).

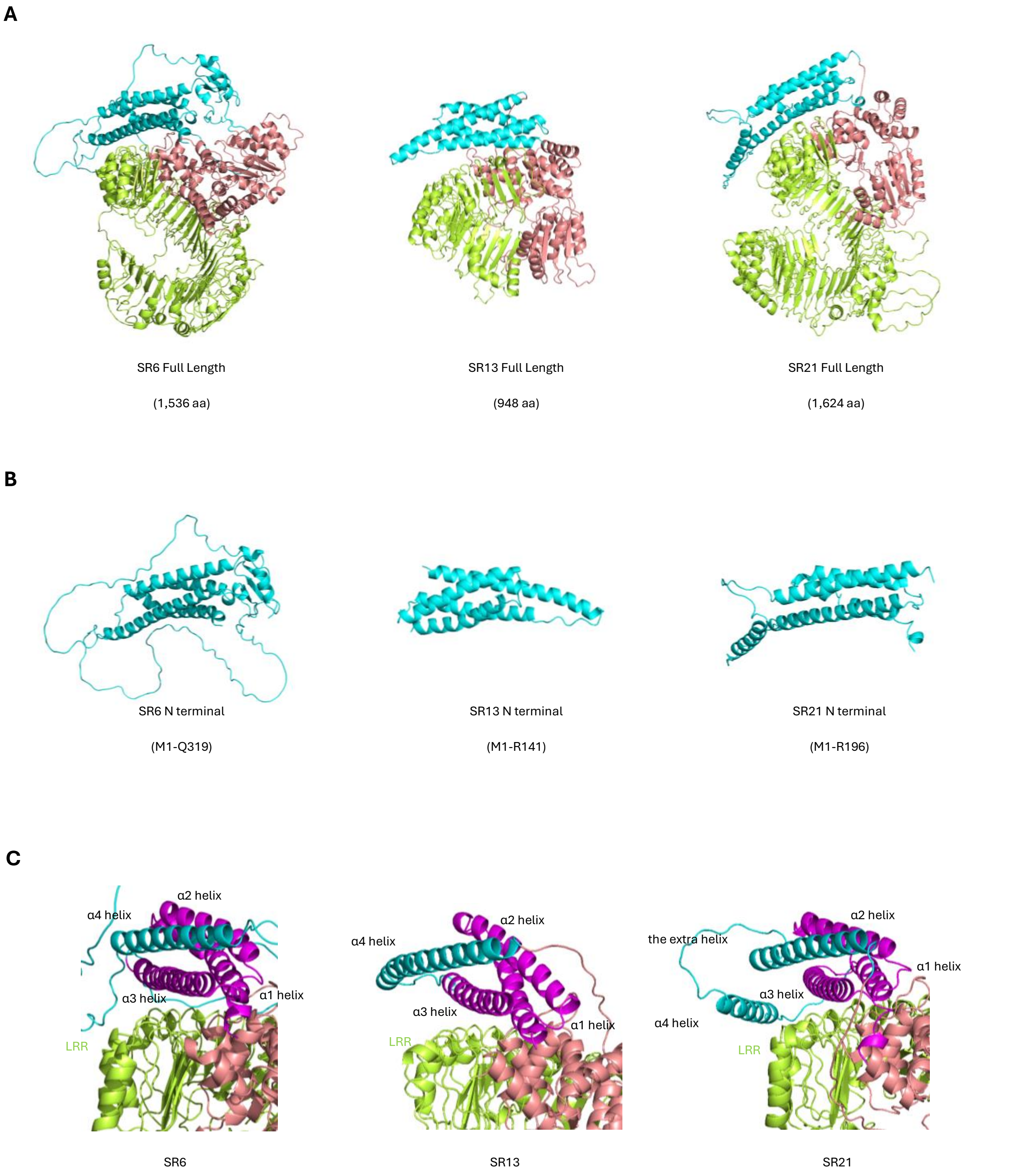

Supplementary Fig. 22. Comparison of the predicted structures of SR6, SR13, and SR21
A: Full length protein structures of SR6, SR13, and SR21 predicted by AlphaFold2. The three conserved subdomains are labelled in cyan (N terminal domains), salmon (NB-ARC domains), and limon (LRR domains); **B:** The distinct N termini of SR6, SR13, and SR21; **C:** The most conserved three helices of each protein are labelled in magenta and in order. The α3 helix of each protein is the closest α helix to the LRR domain (limon color). Source data are provided as a Source Data file.

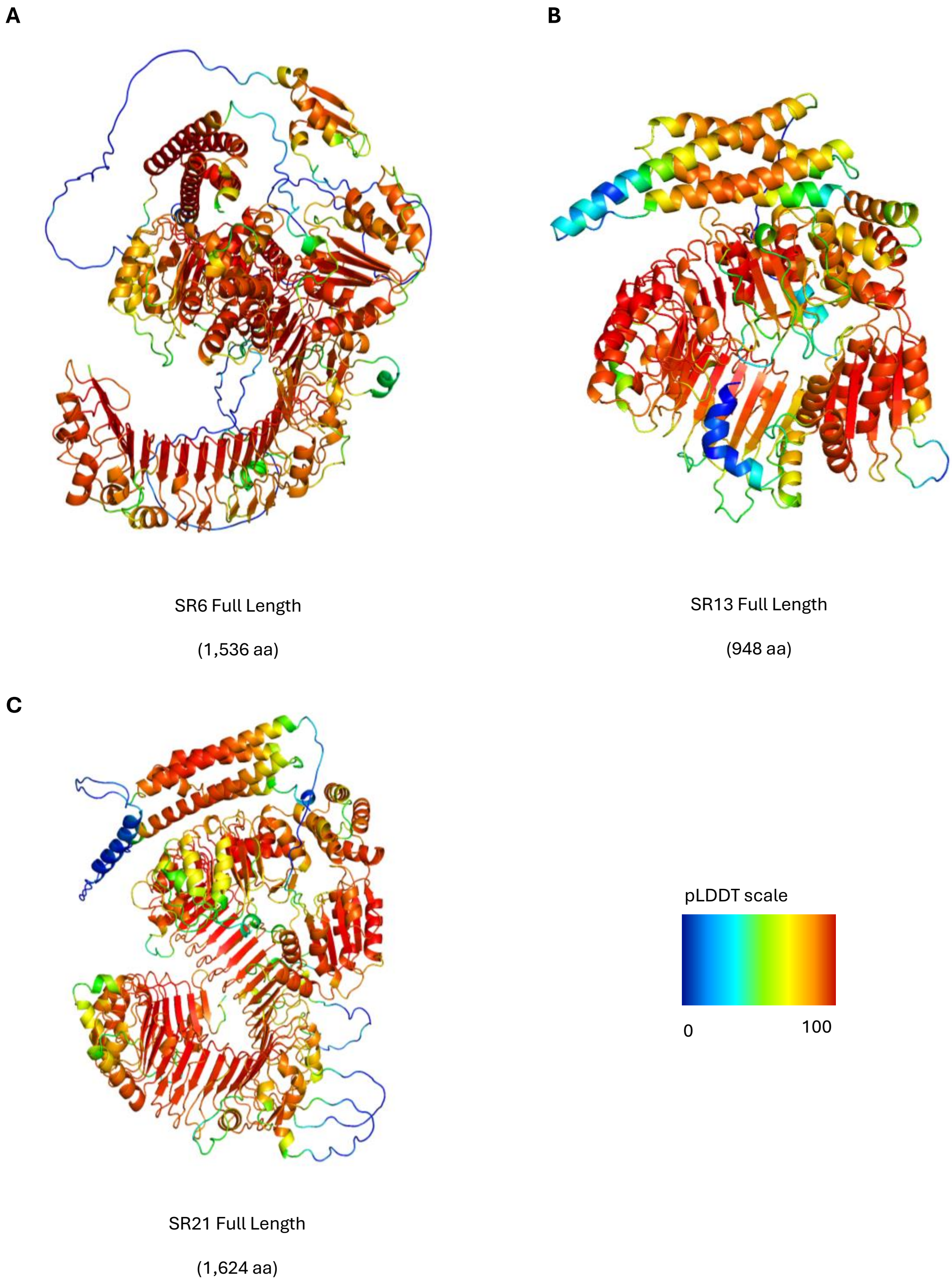

Supplementary Fig. 23. Full-length structures of SR6, SR13, and SR21 predicted by AlphaFold2 shaded with pLDDT values. The pLDDT (predicted Local Distance Difference Test) value of the three predicted full-length proteins are shaded with rainbow colours; red representing high pLDDT value (high confidence) and blue indicating a low pLDDT value (low confidence). The pLDDT values were calculated as ranging from 19.23 to 96.50 for SR6 (**A**), from 26.50 to 96.00 for SR13 (**B**), and 23.95 to 96.73 for SR21 (**C**) in a 0 to 100 scale. Source data are provided as a Source Data file.

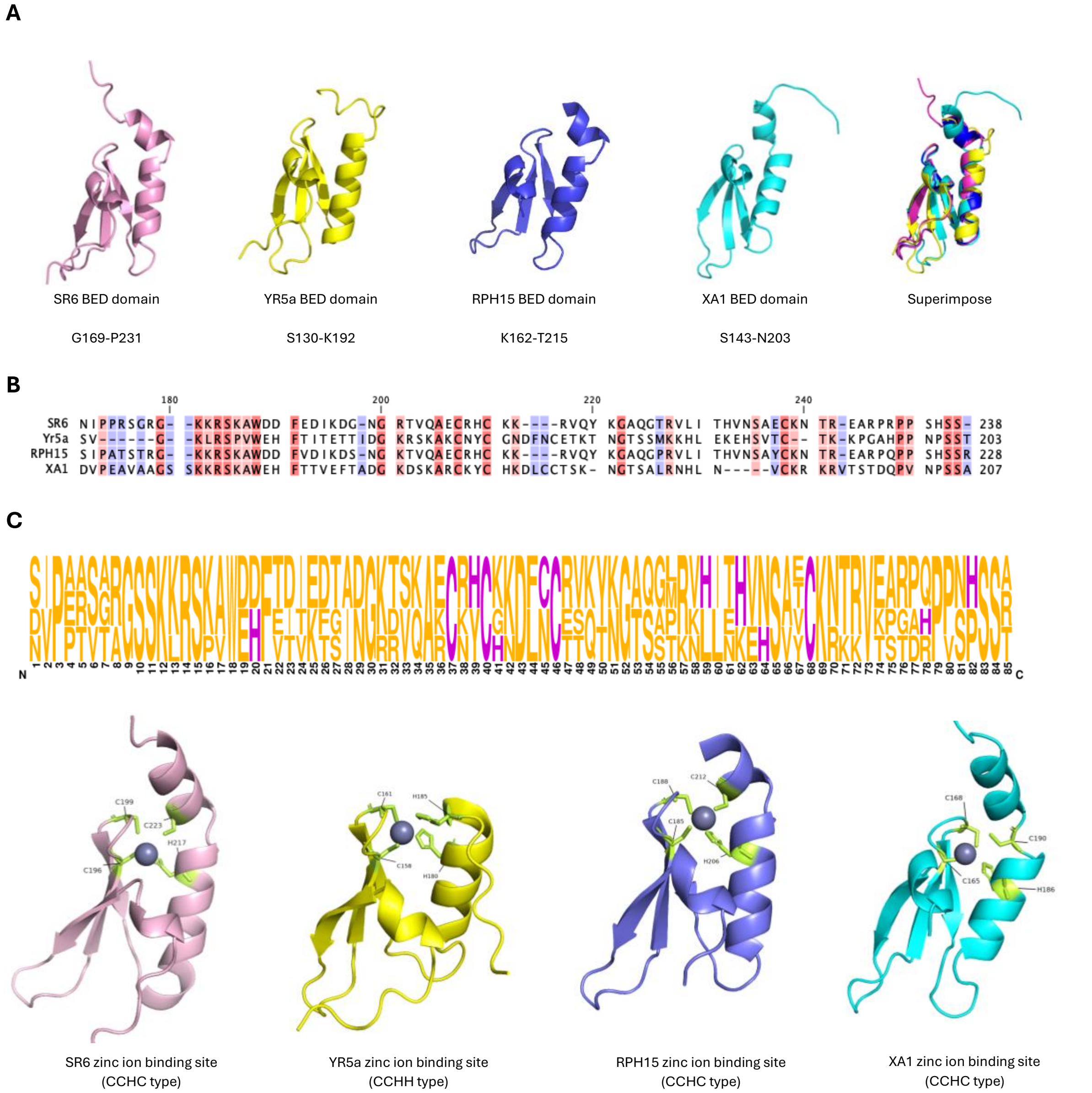

Supplementary Fig. 24. Comparison of the predicted zinc finger BED domains and their zinc ion binding sites in SR6, YR5a, RPH15, and XA1. BED motifs from *Sr6*, wheat stripe rust resistance gene *Yr5*, barley leaf rust resistance gene *Rph15*, and rice bacterial blight resistance gene *Xa1* show high structural similarity (**A**) despite their low similarity in sequence alignment (**B**). **C:** WebLogo figure showing less conservation between the Zinc finger BED domains of SR6, YR5, RPH15, and XA1 with all cysteines and histidines predicted to bind to zinc ions (Zn^{2+}) and resembling a "finger" highlighted in magenta (upper panel). AlphaFold3 predicted the potential zinc ion binding site in SR6, YR5a, RPH15, and XA1, indicating that there are two types of the zinc finger structures among these four proteins, CCHC type (SR6, RPH15, and XA1) and CCHH type (YR5a) (lower panel).

A)

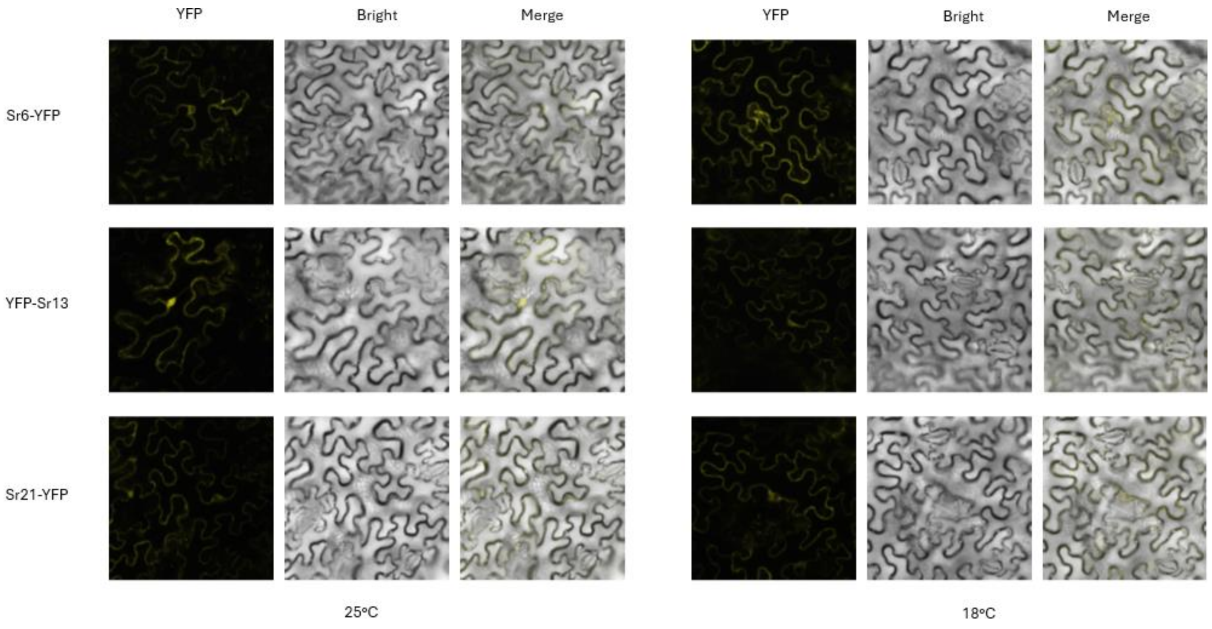

B)

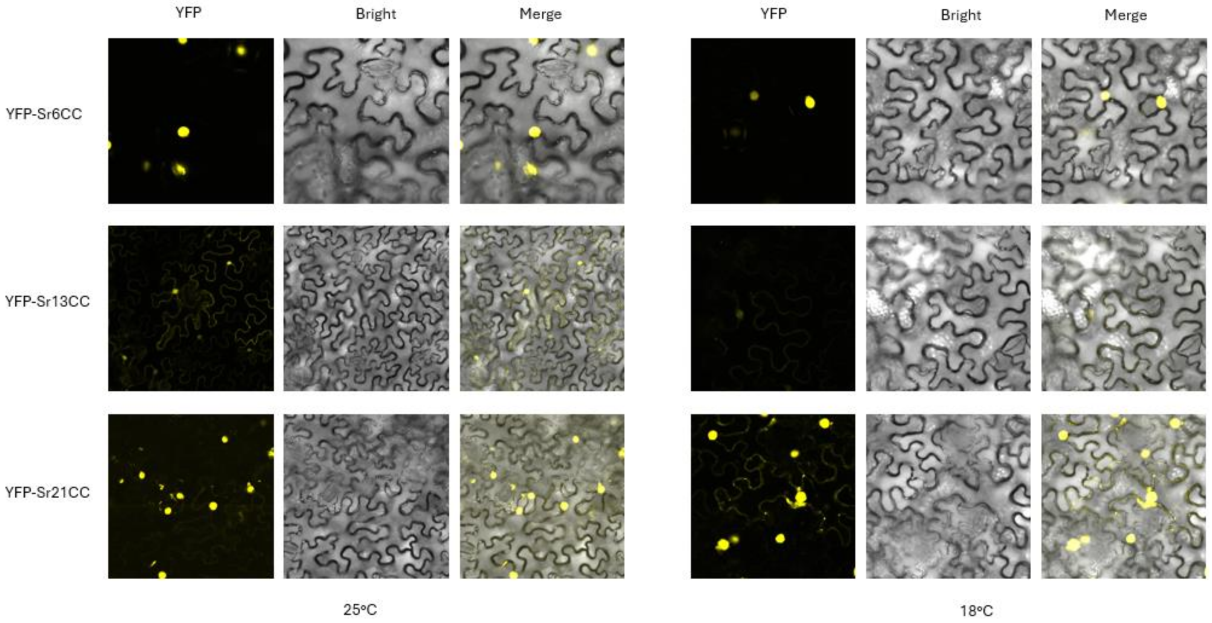

Supplementary Fig. 25. Protein subcellular localisation analysis at two different temperatures. Full-length proteins (**A**) and CC domains (**B**) of SR6, SR13, and SR21 were expressed in *Nicotiana benthamiana* leaves incubated at 25°C (right panels) and 18°C (left panels) for subcellular localization analyses. No differences in protein localization were observed between the two temperatures (left and right panels) or between the different protein constructs (**A**, **B**).

Supplementary Table 1. Polymorphisms observed in point mutants of *Sr6*.

Line ID	Change in CDS	Change in protein	Exon/Intron	Predicted domain
3704-6	C1388T<br>C4572T	P463L<br>synonymous	Exon4<br>Exon4	NB-ARC between motif4 (Kin-2) and motif5 (RNBS-B)
3706-7	C4243T	L1415F	Exon4	LRR
3981-4	G(-)20A	Translation efficiency?	Exon1	5'UTR
4002-6	G1923T	L641F	Exon4	NB-ARC motif8 (Linker)
4190-6	G4309A	E1437K	Exon4	LRR
5047-4	G4466A<br>G4507A	C1489Y<br>E1503K	Exon4<br>Exon4	LRR<br>LRR
5140-5	G3048A	W1016*	Exon4	LRR

Supplementary Table 2. Primers used in this study for PCR and sequencing of *Sr6* candidate.

Description	Forward primer 5'-3'	Forward site	Reverse primer 5'-3'	Reverse site	Annealing temp (°C)	Polymerase	Product length (bp)
STS dominant marker *Sr6STS1*	CCATAGGTGAGGAGCCGTTC	Exon4	CCACGCTATTCCA CATGCC	Exon4	55	GoTaq®	308
STS dominant marker *Sr6STS2*	GGATGGAAACGAATCAAGA TTC	Exon4	CAGGGAATATAGA ACAAAACGTTAAG	Exon4	55	GoTaq®	1029
Amplify NB-LRR region (exon4) of *Sr6* locus	GGATGGAAACGAATCAAGA TTC	Exon4	CCTTTAACTTGCTT GATGCCC	Exon4	66	Phusion®	3807
Amplify CC-BED region (exon1-3) of *Sr6* locus	CATTGGAAGCCCATTTCTCT TAGC	Promoter region	GGTCTGATGGCTG AATTACTGG	Exon4	64	Phusion®	1288
Sequencing primer 1	CGCGATCGTTGAGTGGCTCA C	5'UTR	n/a	n/a	n/a	n/a	n/a
Sequencing primer 2	GTCAATAGCGCCGAGTGTAA G	Exon3	n/a	n/a	n/a	n/a	n/a
Sequencing primer 3	CAGCTATTACTCACCCTTGG AG	Exon4	n/a	n/a	n/a	n/a	n/a
Sequencing primer 4	GCATGCTTGGGTCAGACTCC	Exon4	n/a	n/a	n/a	n/a	n/a
Sequencing primer 5	ATGTCTGGGATGACAAGAAC	Exon4	n/a	n/a	n/a	n/a	n/a
Sequencing primer 6	AGGAGTTAGTCCGTATTTGG	Exon4	n/a	n/a	n/a	n/a	n/a
Sequencing primer 7	TCATCTTCGCTATCTAAAGC	Exon4	n/a	n/a	n/a	n/a	n/a
Sequencing primer 8	TCAGATGACAGCAATGAATC	Exon4	n/a	n/a	n/a	n/a	n/a
Sequencing primer 9	ACGCCTTTGTGAGCTCACTC	Exon4	n/a	n/a	n/a	n/a	n/a
Sequencing primer 10	TGGAATCTCTACAGCTGAAC	Exon4	n/a	n/a	n/a	n/a	n/a
Sequencing primer 11	GAATTTGAGACTTATGCGCA TCTTG	Exon4	n/a	n/a	n/a	n/a	n/a
