# Supplementary material for: Divergent molecular pathways govern temperature-dependent wheat stem rust resistance genes
Source: Nat Commun. 2025 May 28;16:4905. doi: 10.1038/s41467-025-60030-x (PMC12119863; doi:10.1038/s41467-025-60030-x)
Supplement: Supplementary file 3 — Description of Additional Supplementary Files [file 41467_2025_60030_MOESM3_ESM.pdf]

File Name: Supplementary Data 1
Description: qPCR primers used in the study.

File Name: Supplementary Data 2
Description: Gene ontology of jasmonic acid-responsive genes.

File Name: Supplementary Data 3
Description: Gene ontology of the differentially expressed genes in LMPG+*Sr6* under low and high temperatures.

File Name: Supplementary Data 4
Description: Gene ontology of the differentially expressed genes in LMPG+*Sr13* under low and high temperature conditions.

File Name: Supplementary Data 5
Description: Gene ontology of the differentially expressed genes in LMPG+*Sr21* under low and high temperature conditions.

File Name: Supplementary Data 6
Description: Analysis of promoter regions of *Sr6*, *Sr13*, and *Sr21*.

File Name: Supplementary Data 7
Description: R gene accessions used to construct the dendrogram of NLR proteins.
