# Supplementary material for: Divergent molecular pathways govern temperature-dependent wheat stem rust resistance genes
Source: Nat Commun. 2025 May 28;16:4905. doi: 10.1038/s41467-025-60030-x (PMC12119863; doi:10.1038/s41467-025-60030-x)
Supplement: Supplementary file 11 — Reporting Summary [file 41467_2025_60030_MOESM11_ESM.pdf]

nature portfolio

Corresponding author(s): Peng Zhang, Robert McIntosh, Evans Lagudah, Matthew Rouse

Last updated by author(s): Apr 21, 2025

Reporting Summary

Nature Portfolio wishes to improve the reproducibility of the work that we publish. This form provides structure for consistency and transparency in reporting. For further information on Nature Portfolio policies, see our Editorial Policies and the Editorial Policy Checklist.

Statistics

For all statistical analyses, confirm that the following items are present in the figure legend, table legend, main text, or Methods section.

n/a	Confirmed	
☐	☒	The exact sample size (*n*) for each experimental group/condition, given as a discrete number and unit of measurement
☐	☒	A statement on whether measurements were taken from distinct samples or whether the same sample was measured repeatedly
☐	☒	The statistical test(s) used AND whether they are one- or two-sided<br>*Only common tests should be described solely by name; describe more complex techniques in the Methods section.*
☒	☐	A description of all covariates tested
☒	☐	A description of any assumptions or corrections, such as tests of normality and adjustment for multiple comparisons
☐	☒	A full description of the statistical parameters including central tendency (e.g. means) or other basic estimates (e.g. regression coefficient) AND variation (e.g. standard deviation) or associated estimates of uncertainty (e.g. confidence intervals)
☐	☒	For null hypothesis testing, the test statistic (e.g. *F*, *t*, *r*) with confidence intervals, effect sizes, degrees of freedom and *P* value noted<br>*Give P values as exact values whenever suitable.*
☒	☐	For Bayesian analysis, information on the choice of priors and Markov chain Monte Carlo settings
☒	☐	For hierarchical and complex designs, identification of the appropriate level for tests and full reporting of outcomes
☒	☐	Estimates of effect sizes (e.g. Cohen's *d*, Pearson's *r*), indicating how they were calculated

Our web collection on statistics for biologists contains articles on many of the points above.

Software and code

Policy information about availability of computer code

Data collection	No software used

Data analysis	MutantHunter pipeline: https://github.com/steuernb/MutantHunter<br>MuTrigo package: https://github.com/TC-Hewitt/MuTrigo<br>BioKanga package v4.4.2: https://github.com/csiro-crop-informatics/biokanga<br>FGENESH: http://www.softberry.com/berry.phtml<br>CodonCode Aligner v8.0: https://www.codoncode.com/aligner/<br>NLR-Parser: https://github.com/steuernb/NLR-Parser<br>SMART: http://smart.embl.de<br>R package edgeR<br>GeneOntology<br>AgrigoV2<br>ShinyGo<br>MEGA X: www.megasoftware.net<br>AlphaFold3: https://golgi.sandbox.google.com/<br>AlphaFold2: https://colab.research.google.com/github/sokrypton/ColabFold/blob/main/beta/AlphaFold2_advanced.ipynb<br>PyMOL v2.5.5<br>CLC Sequence Viewer v8<br>Weblogo: https://weblogo.berkeley.edu/logo.cgi

For manuscripts utilizing custom algorithms or software that are central to the research but not yet described in published literature, software must be made available to editors and reviewers. We strongly encourage code deposition in a community repository (e.g. GitHub). See the Nature Portfolio guidelines for submitting code & software for further information.

Data

Policy information about availability of data

All manuscripts must include a data availability statement. This statement should provide the following information, where applicable:

- Accession codes, unique identifiers, or web links for publicly available datasets
- A description of any restrictions on data availability
- For clinical datasets or third party data, please ensure that the statement adheres to our policy

The data generated in this study are openly available in NCBI, the mRNA sequence of Sr6 have been deposited at NCBI GenBank with accession number PP949235. Sequencing read data for MutRenSeq is deposited at DDBJ/EMBL/GenBank under BioProject PRJNA1127689 under accessions SRX28382285-SRX28382292. Other data generated in this study are provided in the Supplementary Information/Source Data files. Source data are provided with this paper.

Research involving human participants, their data, or biological material

Policy information about studies with human participants or human data. See also policy information about sex, gender (identity/presentation), and sexual orientation and race, ethnicity and racism.

Reporting on sex and gender	None
Reporting on race, ethnicity, or other socially relevant groupings	None
Population characteristics	None
Recruitment	None
Ethics oversight	None

Note that full information on the approval of the study protocol must also be provided in the manuscript.

Field-specific reporting

Please select the one below that is the best fit for your research. If you are not sure, read the appropriate sections before making your selection.

☒ Life sciences ☐ Behavioural & social sciences ☐ Ecological, evolutionary & environmental sciences

For a reference copy of the document with all sections, see nature.com/documents/nr-reporting-summary-flat.pdf

Life sciences study design

All studies must disclose on these points even when the disclosure is negative.

Sample size	All the information regarding sample size were specifically described in the manuscript.
Data exclusions	No data were excluded from the analyses.
Replication	Mutant plants were identified in M2 generation and were confirmed by progeny test in M3 generation.

	For qPCR, there are both biological replicates and technical replicates.
Randomization	When phenotyping plants, we randomized mutant, wild-type, and control plants as is normal practice.
Blinding	Blinding was performed when phenotyping the mutant lines for their resistance to Pgt, candidate gene confirmation by wheat transformation, qPCR for determining transgene expression. It is not performed when carrying out RenSeq, Sequencing analysis, phylogenetic analysis, protein structure predictions as required by the experiments.

Reporting for specific materials, systems and methods

We require information from authors about some types of materials, experimental systems and methods used in many studies. Here, indicate whether each material, system or method listed is relevant to your study. If you are not sure if a list item applies to your research, read the appropriate section before selecting a response.

Materials & experimental systems

n/a	Involved in the study
☒	☐ Antibodies
☒	☐ Eukaryotic cell lines
☒	☐ Palaeontology and archaeology
☒	☐ Animals and other organisms
☒	☐ Clinical data
☒	☐ Dual use research of concern
☐	☒ Plants

Methods

n/a	Involved in the study
☒	☐ ChIP-seq
☒	☐ Flow cytometry
☒	☐ MRI-based neuroimaging

Plants

Seed stocks	Chinese Spring*5/Red Egyptian 2D, Chinese Spring, and Westonia (PBI) are maintained at the Plant Breeding Institute, University of Sydney; Westonia (CSIRO) is maintained at CSIRO Canberra; LMPG, LMPG-Sr6, LMPG-Sr13, and LMPG-Sr21 are maintained at the Cereal Disease Laboratory, USDA-ARS, St. Paul.
Novel plant genotypes	2,700 seeds of wheat line Chinese Spring*5/Red Egyptian 2D were mutagenized with EMS. Seven independent mutants were identified in M2 generation using the Sr6-avirulent race 21-2,3,7.
Authentication	The seven mutants obtained were progeny tested and confirmed at the M3 generation.
