# Supplementary material for: Divergent molecular pathways govern temperature-dependent wheat stem rust resistance genes
Source: Nat Commun. 2025 May 28;16:4905. doi: 10.1038/s41467-025-60030-x (PMC12119863; doi:10.1038/s41467-025-60030-x)
Supplement: Supplementary file 12 — Source Data [file 41467_2025_60030_MOESM12_ESM.zip › Source Data_Suppl Figs 22, 23.zip!/Sequence used for structure modelling.fa]

>Sr6aa
MEDAYLVPAILWLVQTILGTLGIRELDSWIRQAGLQDDIENLRSELEVLQPVASDAKGMALRSARLAKSLADLKRFLYDADDLIDELDYFRLQHQVEGVTNTVSISQEPLPSSSNAHNRFASDGVTQPCTAAATTTAVTLQEPEGMHEAAPVDHAGTLRANDNIPPRSGRGKKRSKAWDDFEDIKDGNGRTVQAECRHCKKRVQYKGAQGTRVLITHVNSAECKNTREARPRPPSHSSIDDADENAIPVAASNSAIRPRMETNQDSTQITAAITHPWSKDEISSRIREITRQLQGIRGDVTKDLSMLGSDSGASSGHHQSTASDPCRRTSSLLQGKVYGRDKEKSSIIKLMTAGKSDSVTVVPIVGIGGTGKTALAQSVYNDPKVQDHFDHIIWIWVSNIFDEMRISREMLDVVPREKHEGLCSLDKLQQVLKTHIESKRVLLILDDVWDDKNNRRLDLILAPFKSNNANDSVILVTTRDLSVAKRIGTTEPVVLGTLENEDLWLLLKERAFGDENYKGHGNLTNIGRKIAWKLKGNPLAAVTAGELLRVDLTVDHWIKILKDEDWKLLGLSGGIMSSLKFSYDQLPYHLHQCLTFCSIFPDNHEFPDEELVRIWISQGFVKQSNPSKRLEDIGMDYLSDLVNLSYFQRVGRKDSYVVCGLMHEFSRIVSRTDCAIIDGLECTDILPTVQHLSVECKKDQHGNVSWSSKFKEDLRKTVTSVRKLRTLLLIGQYDCSFFQSFEDIFQKAHYIRLLQISATFQDFNSLVRSSVNPTHLRYLKLTVNEVQGDLPQVLSNFCHLQVLDVGSNTHLDVAHGMDNLVSLRHLVVASIYRIGKLTSLQTLSNFSVQDSNGFRIIELQSMNDLAQLMISQLQNVKTLEEACGAGLRYKRHLEKLHLSWNMSDVESDSDGSNESDSDGSNESDSDDSNESDSDGSNESDSDDSNESENVMSSEPSTSKETEECLPTEVLEGLKPHYNLKHLQITGYNGAASPSWLARNISVTCLQTLYLNGCGEWRILPSLRGLPFLANLKLRNMPKVEEVWVLSLKKLVLVKMPKLERCSCVSLRDLNSCLRVLKIKHCPALKVFDLFGNGHEFKIEQKSWLPRLCELTLKNCRLLVVPYPLPSASTVCSISVSRVSKFPTIEVWSCILTIGEEPFVGRVPFSEDYYNKFNDELFVLDGNILAFHNLRFLTQLIIGGCQKLTSISLKGLSQLESLKTLKIKGCARLSGLDAPPEHALPSLEYLEIGACGIAWEWLSLILQHAPALTELDLWDCPKLESLQLNSCTTLQKLSVDIFESLGTLELHFCTALEDLFIREAAVCVLEGSQSLRKLRLHGSHYLESLKLHSCTALEELDIRSCTSLCTLEGFQSLGSLRRLYLRDCPGLHSFLEGLPEQDYNLCPRLEFLDISGFSFLATPFCKQLTSLQRLVFCGLDGEKTEEGLTDEQERGLLLLGSLTELEFETYAHLEYLPASLHRLRSLQVLEISFCPSISGLPDLPPSLEELRIFHGSEELKHQSISRASSKLKVKIDGQYVN
>Sr13aa
MEAALVTVATGVLKPVLGKLATLLGDEYKRFKGVRKEIRSLTHELAAMEAFLLKMSEEEEDLNVQDKVWMNEVRELSYDMEDAIDDFMQSVGDKEEKPDGFIDKIKSSLGKLGNMKARHRIGKEIQDLKKQIIEVGDRNARYKGREIFSKAVNVTVDPRALAIFEHASKLVGIDEPKAELIKLLTDKDGVASTQQQVKMVSIVGSGGMGKTTLANQVYQELKEKFKCKAFISVSRNPDMTNILRTLLSEVGCQDYADTEAGSIQQLIRKITDYLAEKRYIIVIDDIWDVKTWDVIKCAFPMTRCGGVIITTTRLSDVARSCHSSIGGHIYNIRPLNMEHSRQLFHRRLFSSEEDCPSSLVKVSNQILEKCDGLPLAIIAIAGLLANTGRSEHLWNQVKDSIGRALERNPNVEVMIKILSLSYFDLPPHLKTCLLYLSIFPEDSIIEKKTLISRWIAEGFIQKEGIYTAYEVGVRCFNELINRSLIQPVKKDDYRGKSCRVHDIILDFIVSKSIEENFVTFAGVPSLTTVTQGKVRRLSMQVEGKGDSILPMSPILSHVRSFNVFRNRVNIHSTMEFRHLRVVDFNDSLLENHHLANVGRLLQLRYLSIYMTAVSELPEQIGHLQCLEMLDIRYTMVSELPASIVNLGKLAHLLLGSEDTCVKFPDGIAKMQALETLDEVDASKQSYNFLQGLGRLKNLRKLHIDYHDVAQEDKEVIASSLGKLCTQNLCSLTMRGNDDDDFLLNTWCTSPPLNLRKLVIWGCIFPKVPHWVGSLVNLQKLRLHVGKEIRHEDICILGALPALLTLGLKGMQKQPSCEDGRLAVSGEAGFRCLRKFKYCRWGDRMDLMFTAKCMPKLEKLKIIFYRHAQDEAPIIPAFDFGIENLSRLTTFKCHLGCRPMATRTFDAVKASLDRVVRAHPNHLTVIFSYPLRTSDMTYTFHDCYMRSQD
>Sr21aa
MSGLLGTVVDATIGWLVQSILDSFFTGRMEAWTREIGLAEDVEKLKFQMRDVQMVLAAAEGRRIDNMPLALSLDGLRELLYDSEDVMDELDYYRLEQQITEGKGCSAPTGTNLEGSYVSSSTLSSVVKSVCSATSQITDWISRGRKRKREEEGPAHCNMLPFEIKDSISKRINVIVNLLCSSSNSVKGVLQLENLRTMATSSKSQNIARDHRMTTSVPIEYKVYGRDAERDEIIDLLIKGGSSDLNVLPVVGSGGIGKTTLVRYVYHAKRIKDHFDLLIWVCVSTNFDVVGLTLQILDHVCEDRPYEKKSSLNKLQEILQENIRNKRFLLVMDDMWEEKDRGGWIKLLAPLKSNKVKGCMVLATTRTKSVAKMIGTMDEITLTGLDEKEFWLFFKACAFRNDNREHHPSLQSIGKQIAKALKGFPLAAQSVGALLSTEVSYQHWTTVRDKWKSLQGYDDDILPILKLSYDYLPVYLQRCFSYCSLYPEDYGFDGKELVHAWISQNFVQCKDPTIRLEETGHEYLEKLVDLGFFQKDGSHYVMHDLMHALAGMVSSNECATIDGLKSGAIRASVRHLSIIITDYDKDEHVSNSSEKFDKILQKVSWHKLRTLMLFGRSSIHLSGPLRTLCNVAKCLRLLSVTGADISSIYNSSNLFHLRYISAYRVSNPAFRQALTRCYHLQVLDVGISGNLDVPTDMNNLVNLRHLIAHEKVHHAIDCVSNMTSLQELKFKVQNVGSFEIGQLQSMNELVSLGVSQLENVKTKEEAWAAMLTHKEYLETLFLSWENSSMSLQPEAAEDVLDGLQPHQNLKTLEITGYGGAISPTWLSSAFSVTSLQILHLEECREWQILSTHGMHSLRKLTLIRMLNLMELSVPSLVELILIGMPKLKKCTGSYGMDLTSHLSVLMIKNCPQLNELTLFQSYSSFDAEQKSWFPSLSKLSIGQCPHIINNWPILPLREMGALKELELMDLHVVRVSVPSLEKLVLTKMPILEYCSSLTTSPPLQSSPPEGDQNELVSNLRRLTIHDCPCLIVSHPLPPSALISELSISGVSTLPTMRINWRHFTIESNELCMLDESILAFHNLRGITLFQIRSCPNLISLSSESFSQLIALETLSIHECPNLTMSNIMPEVVQENSTSTSSLVPPSLKRVNISTCGVTGRWLSQLLSHSQSLEELLLTGCPQIKFLSISQPRETEGTSSLVSAVTTSAQDEHELKLPYNLLCSLKALWIQLSLDLEFCGGYRDFAGFTSLMDLVLFGCPKLVSSLVGETKDDGTMEVGLLPPALEKVTISPLPESLRSFTPQGLLHLKELSLFYGPCLKSVQLHSCTSLVELGITGCVQLAVLEGLQFLTSLRSLDIEMSPELSSAWDLKLQEQEQGGNQIQLLPPSLDKLEIRALTDSVQSHLLSCLPAITKLAIGRSPELTSLQLGCCTALKELEIEKCDSLESIEGLQFCRNLKSLRVFNSPGLASFLELVSHQQGASVTLSGLQTLEASDGSVLSTHFCKQLTSLRLLQFGSEASEGRGESLVSLTEEQEGALQLLTSLQELQFRRCQNLLSLPANLNSLTSLETLSIIHCKSITRLPDMGLPTSLRYLKLFYCSEELAVQCRIVATEKLRVRIDRQDVN
